# Transcriptional re-programming of liver-resident iNKT cells into T-regulatory type-1-like liver iNKT cells involves extensive gene de-methylation

**DOI:** 10.3389/fimmu.2024.1454314

**Published:** 2024-09-09

**Authors:** Javier Montaño, Josep Garnica, Jun Yamanouchi, Joel Moro, Patricia Solé, Debajyoti Mondal, Pau Serra, Yang Yang, Pere Santamaria

**Affiliations:** ^1^ Institut D’Investigacions Biomèdiques August Pi i Sunyer, Barcelona, Spain; ^2^ Department of Microbiology, Immunology and Infectious Diseases, Snyder Institute for Chronic Diseases and Hotchkiss Brain Institute, Cumming School of Medicine, University of Calgary, Calgary, AB, Canada; ^3^ Department of Biochemistry and Molecular Biology and Snyder Institute for Chronic Diseases, Cumming School of Medicine, University of Calgary, Calgary, AB, Canada

**Keywords:** iNKT cell, αGalCer/CD1d-coated nanoparticles, liver iNKT cell, iNKT cell re-programming, gene de-methylation, liver iNKT-regulatory type-1 (LiNKTR1) cells, epigenetics

## Abstract

Unlike conventional CD4+ T cells, which are phenotypically and functionally plastic, invariant NKT (iNKT) cells generally exist in a terminally differentiated state. Naïve CD4+ T cells can acquire alternative epigenetic states in response to different cues, but it remains unclear whether peripheral iNKT cells are epigenetically stable or malleable. Repetitive encounters of liver-resident iNKT cells (LiNKTs) with alpha-galactosylceramide (αGalCer)/CD1d-coated nanoparticles (NPs) can trigger their differentiation into a LiNKT cell subset expressing a T regulatory type 1 (TR1)-like (LiNKTR1) transcriptional signature. Here we dissect the epigenetic underpinnings of the LiNKT-LiNKTR1 conversion as compared to those underlying the peptide-major histocompatibility complex (pMHC)-NP-induced T-follicular helper (TFH)-to-TR1 transdifferentiation process. We show that gene upregulation during the LINKT-to-LiNKTR1 cell conversion is associated with demethylation of gene bodies, inter-genic regions, promoters and distal gene regulatory elements, in the absence of major changes in chromatin exposure or deposition of expression-promoting histone marks. In contrast, the naïve CD4+ T cell-to-TFH differentiation process involves extensive remodeling of the chromatin and the acquisition of a broad repertoire of epigenetic modifications that are then largely inherited by TFH cell-derived TR1 cell progeny. These observations indicate that LiNKT cells are epigenetically malleable and particularly susceptible to gene de-methylation.

## Introduction

iNKT cells develop in the thymus and exist as five major subsets with distinct cytokine and transcription factor expression profiles (1). Upon thymic differentiation, iNKT cell subsets migrate to peripheral tissues with distinct frequencies, where they persist as terminally differentiated cells (1–9). However, the mechanisms responsible for the transcriptional, phenotypic and functional stability of peripheral iNKT cell subsets remain unclear.

Differentiation of thymic iNKTs into distinct iNKT cell subsets is known to involve changes in chromatin accessibility. For example, during the double positive (DP) to stage 0 transition, loci such as *Zbtb16*, *Tbx21* or *Ifng* become differentially accessible and upregulated (10). Likewise, the enhancers of genes that are differentially expressed and regulated by iNKT cell subset-specific transcription factors are enriched for acetylated H3K27, an expression-promoting histone mark (11). As a result, peripheral iNKT1, iNKT2 and iNKT17 cell subsets display significant differences in the genome wide distribution of open chromatin regions, particularly at loci that are differentially expressed among these three subsets (11–14). These differences are maintained across tissues, including thymus, liver, lung and spleen, consistent with the thymic origin of peripheral iNKT subsets. Notwithstanding the general similarities in the distribution of accessible chromatin sites of specific iNKT cell subsets residing in different tissues, iNKT cells residing in certain foreign antigen-rich organs, such as the lung and the small intestine, display unique open chromatin signatures that are probably acquired shortly after organ colonization by thymic-derived iNKT cells (15).

Interestingly, the open chromatin landscape of the splenic alpha-galactosylceramide(αGalCer)-reactive iNKT cell pool undergoes significant changes upon αGalCer challenge, leading to the formation of a follicular T helper cell-like iNKT cell subset (iNKT_FH_) that displays increased *Il21* and *Pdcd1* locus accessibility and gene expression, and decreased accessibility of loci regulated by the iNKT2- and iNKT17-associated GATA3 and RORγt transcription factors, respectively (15). αGalCer exposure also changed the distribution of open chromatin sites in splenic non-iNKT_TFH_ cells towards an iNKT_eff_-like subset that upregulates Granzyme A and B (15). Collectively, these observations suggest that peripheral iNKT cells may be epigenetically malleable.

We have recently shown that intravenous delivery of NPs displaying αGalCer/CD1d complexes can trigger the differentiation of liver-resident iNKT (LiNKT) cells into a novel LiNKT subset that acquires T-regulatory type 1 (TR1)-like transcriptional, phenotypic and functional properties, including an ability to orchestrate the formation of a local immunoregulatory cell network that blunts various forms of liver autoimmunity (16). This outcome mirrored that obtained in mice treated with NPs coated with mono-specific autoimmune disease-relevant peptide-major histocompatibility complex class II (pMHCII) molecules, which promotes the transdifferentiation of T-follicular helper (TFH) cells into TR1 CD4+ T cells with a similar transcriptional signature and immunoregulatory properties (17–21). For example, both subsets (TR1 and LiNKTR1) co-express the immunoregulatory cytokines IL-10 and IL-21, the transcription factors c-MAF, T-BET, IRF4 and NFIL3 and the checkpoint inhibitors LAG-3, PD-1, CTLA-4 and TIGIT, among others (16, 22, 23).

The transcriptional, phenotypic and functional similarities between αGalCer/CD1d and pMHCII-NP-induced LiNKTR1 and TR1 cells, respectively, offered a unique opportunity to investigate whether the epigenome of LiNKTs is plastic or imprinted, as compared to the epigenomes of naive CD4+ T and TFH cells, respectively. Through a combination of bulk and single-cell transcriptional and epigenetic studies, we have confirmed that the naïve CD4+ T cell–to–TFH conversion preceeding TR1 cell generation is accompanied by major changes in chromatin structure, transcription-enhancing histone deposition maps, and de-methylation of gene bodies and gene regulatory sequences (24). Such epigenetic changes are reminiscent of those described during T-helper subset (i.e. Th1/Th2/Th17) or FoxP3+ Treg cell specification, which involve DNA de-methylation and deposition of expression-enhancing (and removal of repressive) histone marks on regulatory regions of differentially upregulated genes (25–34). In contrast, the TFH-TR1 cell transdifferentiation process is largely dissociated from these epigenetic modifications, indicating that the TR1 gene expression program is genetically imprinted at the TFH cell stage (24).

Here, we show that LiNKTR1 cell generation in response to αGalCer/CD1d-NP engagement is associated with treatment-induced hypo-methylation of bodies and regulatory elements of upregulated genes, albeit in the absence of significant changes in chromatin exposure or deposition of expression-promoting histone marks.

## Results

### The αGalCer/CD1d-NP-induced LiNKTR1 cell subset is polyclonal

We have previously shown that autoimmune disease-relevant pMHCII-NPs trigger the expansion of cognate (pMHCII tetramer+) TFH-like cells, followed by BLIMP-1-dependent re-programming of these expanded TFH cells into TR1 cell progeny (22). Indeed, TCR tracing experiments have shown that there is extensive TCRαβ clonotype sharing between the tetramer+ TFH and TR1 cell sub-clusters. These and other data provided direct evidence for a lineage relationship between the TFH and TR1 cells arising in response to pMHCII-NP treatment and confirmed that TR1 formation was preceded by cognate TFH cell expansion (22).

To ascertain whether αGalCer/CD1d-NPs operate via a similar mechanism (e.g., by triggering the proliferation of LiNKT cells followed by their differentiation into LiNKTR1 cells), we reconstructed the TCRαβ pairs expressed by individual liver-resident αGalCer/CD1d tetramer+ cells isolated from both αGalCer/CD1d-NP-treated and untreated NOD mice (**Supplementary Figure 1**). We sequenced the RNA of a total of 528 cells (277 from untreated and 251 from treated mice) via single cell SmartSeq2. Of these 528 cells, 343 (181 from untreated and 162 from treated mice) expressed productive TCRα and TCRβ rearrangements (Datasheet 1).

**Figure 1:**
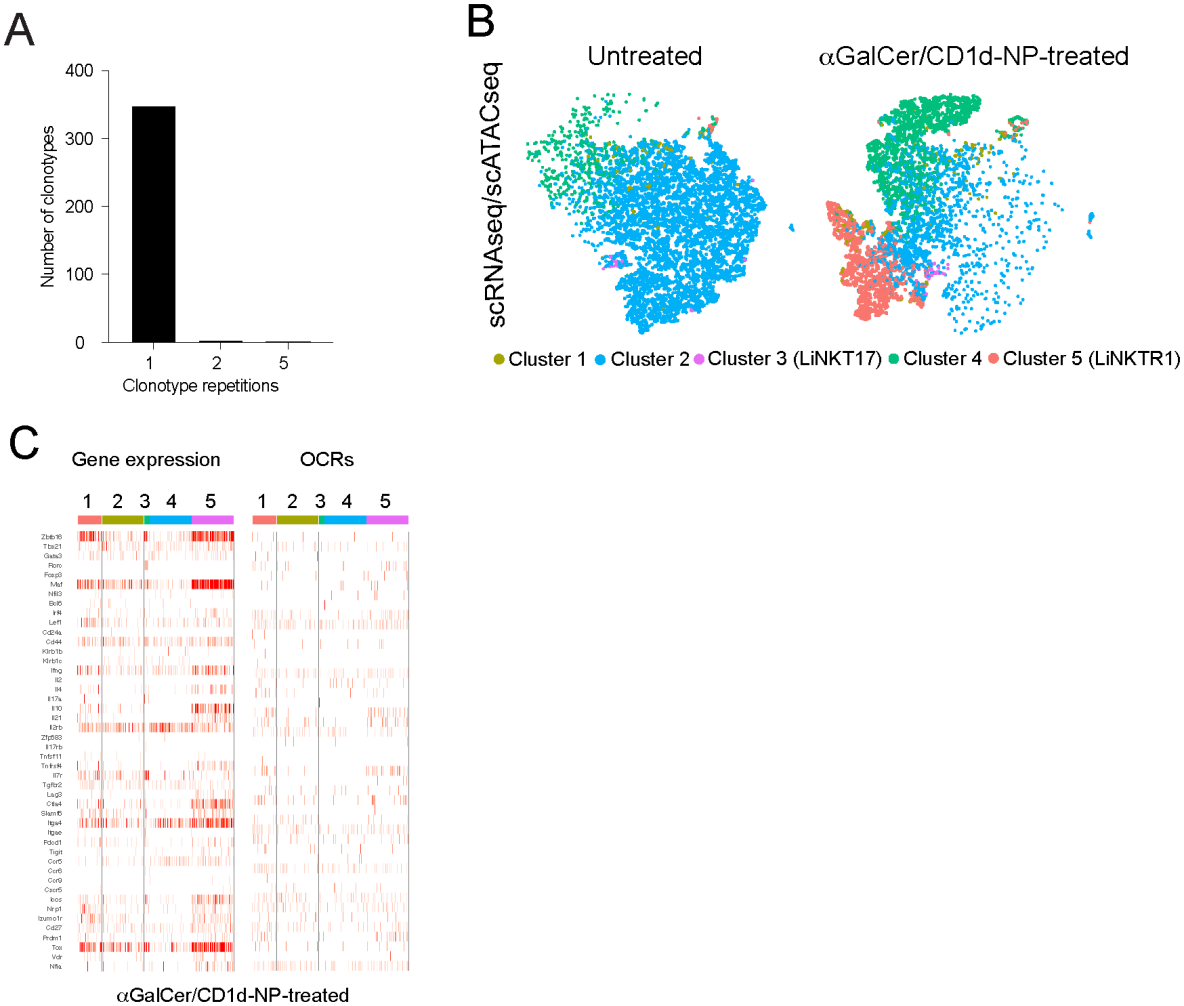
Treatment induced changes in open chromatin and gene expression at the single cell level. **(A)** Clonotypic diversity of the LiNKT cells from αGalCer/CD1d-NP-treated and untreated mice. Data correspond to TCRαβ sequences expressed by individual cells as determined via SMARTseq2. Most clones (325/343; 94.75%) cells expressed unique TCRαβ pairs. There were only two small clonal groups of 5 cells each (10/343 cells; 2.91%) and 4 clonal groups of 2 cells each (8/343 cells; 2.33%) that shared TCRαβ pairs. See main text for additional details. **(B)** tSNE plots for the scMultiomes of LiNKT cells from control (left) vs. αGalCer/CD1d-NP-treated mice (right). **(C)** Heatmaps comparing the normalized expression levels (scRNAseq, left) and intensity of open chromatin regions (OCRs) (scATACseq, right) data for a selection of 46 LiNKT relevant genes (listed in **Supplementary Table 1**). Data correspond to LiNKT cells sorted from mice treated with vehicle (n=5 8wk-old male NOD mice; ~300,000 cells) or αGalCer/CD1d-NPs (n=4 8wk-old male NOD mice).

As expected, the vast majority of the productive TCRα sequences (333/343; 97.08%) used the prevalent TRAV11 and TRAJ18 elements and a significant fraction of these rearrangements encoded identical CDR3 sequences. In contrast, although most TCRβ rearrangements used the TRBV13-2 (n=196), TRBV13-1 (n=35) and TRBV1 (n=18) elements, their CDR3 regions were diverse. That is, most LiNKT cells from both untreated and treated NOD mice, including all the cells belonging to the αGalCer/CD1d-NP-induced LiNKTR1 cell sub-cluster 5, corresponded to unique clonotypes that lacked “twins” in other sub-clusters (n=325/343; 94.75%) (Datasheet 2). Exceptions included two clonal groups of 5 cells each in the LiNKT cell sub-cluster 4 from αGalCer/CD1-NP-treated mice and 4 clonal groups of 2 cells each in: (i) cluster 4 from untreated mice; (ii) cluster 4 from treated mice; (iii) clusters 2 and 4 from untreated mice; and (iv) cluster 2 from untreated mice (**Figure 1A**) (Datasheet 2).

Collectively, these results indicate that the αGalCer/CD1d-NP-induced re-programming of LiNKT into LiNKTR1 cells, unlike the case for pMHCII-NP-induced TFH-to-TR1 cell re-programming, is neither preceded nor accompanied by LiNKT or LiNKTR1 cell proliferation. That is, this process cannot be accounted for by treatment-induced expansion of a pre-existing sub-cluster of LiNKTR1 cells and likely involves a direct conversion of LiNKT cells into LiNKTR1 cells.

### Treatment induced changes in open chromatin and gene expression at the single cell level

**Figure 2:**
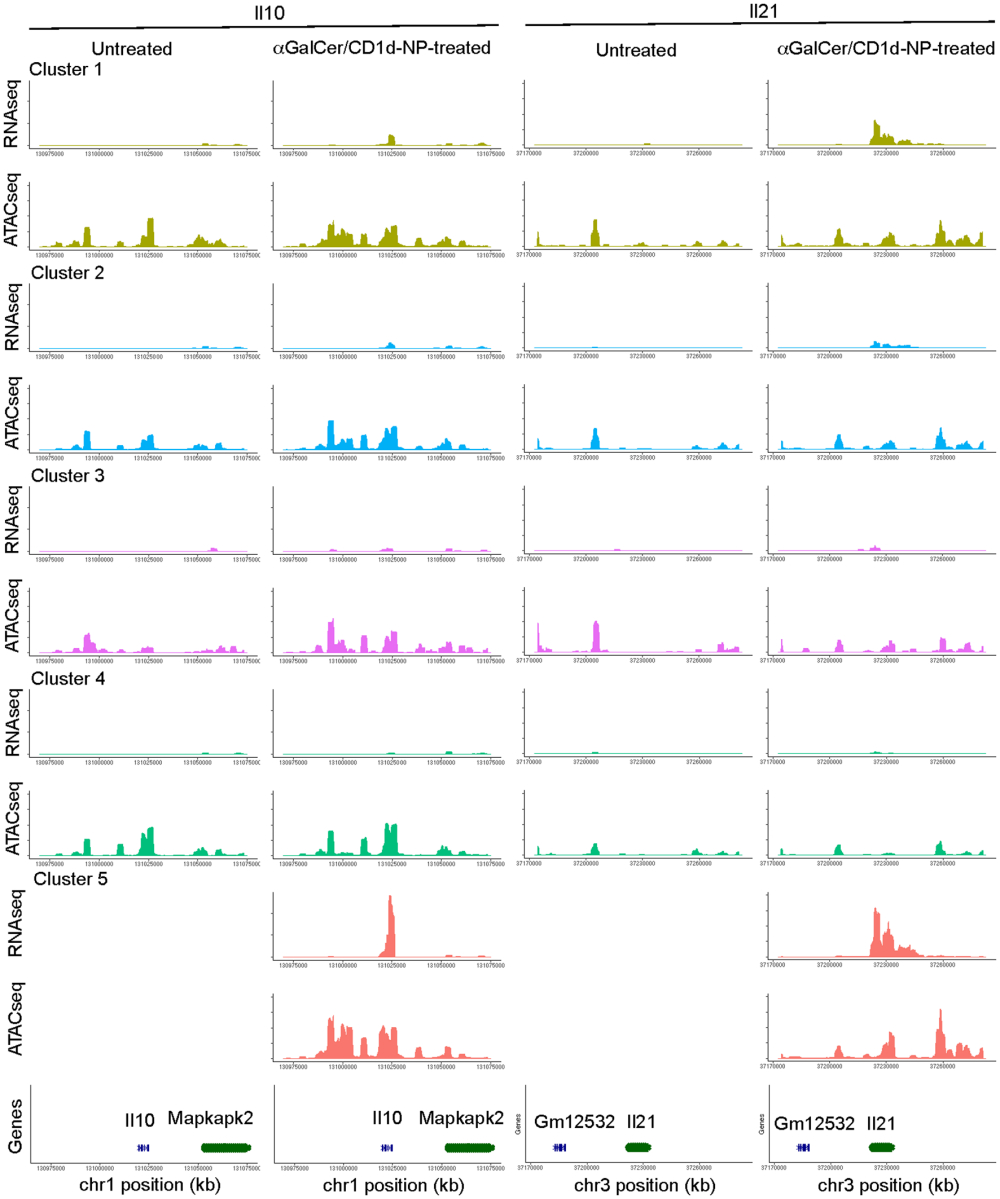
RNA and OCR tracks for two representative sub-cluster 5 genes: *Il10* and *Il21* (αGalCer/CD1d-NP-induced). Chromosome tracks for *Il10* and *Il21* displaying gene expression and open chromatin data for each of the LiNKT cell sub-clusters identified in control vs. αGalCer/CD1d-NP-treated mice.

We next sought to confirm the above observations by comparing the two-dimensional multiomes (scRNAseq+scATACseq) of the LiNKT cells from αGalCer/CD1d-NP-treated and untreated NOD mice. The data obtained from this multiome experiment (using nuclei only, as opposed to our previously published scRNAseq data, which used whole cells) confirmed the presence, in untreated mice, of the 4 LiNKT cell sub-clusters that had been identified previously. Weighted-nearest neighbor’ (WNN) analysis of multiomic data revealed a significant degree of overlap between all four clusters (**Figure 1B**, left). In αGalCer/CD1d-NP-treated mice, there was a decrease in the size of cluster 2 and an increase in the size of cluster 4, and a *de novo* appearance of the LiNKTR1 cluster 5, as previously reported (16). Whereas the scMultiomes of clusters 3 and 4 did not undergo significant changes in response to treatment, the scMultiome of cluster 2 cells became similar to that of cluster 5 (**Figure 1B**, right). Nevertheless, comparison of scRNAseq and scATACseq data for iNKT-relevant genes, indicated that the 5 LiNKT cell clusters are epigenetically similar, despite being transcriptionally different (**Figure 1C**). **Figure 2** compares the RNA expression and chromatin accessibility genome tracks for two representative cluster 5 (LiNKTR1) genes: *Il10* and *Il21*. Although the genomic tracks shown in **Figure 2** suggest that *il10* and *il21* may undergo chromatin remodeling in cluster 5 cells, thorough statistical analysis involving signal background for each condition and P value adjustment did not reveal differential chromatin accessibility around these loci when comparing cluster 5 cells to cells from the other clusters. Collectively, these data indicate that the various αGalCer/CD1d-specific LiNKT cell sub-pools share a remarkably similar scATACseq landscape.

### Treatment-induced upregulation of LiNKT cell gene expression is largely dissociated from *de novo* changes in H3K4me3 and H3K27ac marks

Among the many epigenetic modifications of histones that regulate gene expression, deposition of H3K4me3 and H3K27ac play key roles. The addition of 3 methyl groups to the Lys4 of Histone 3 (H3K4me3) marks transcriptional start sites (TSS) in active promoters. The acetylation of Lys27 in Histone 3 (H3K27ac) marks active enhancers and/or promoters of transcriptionally active genes (35).

**Figure 3:**
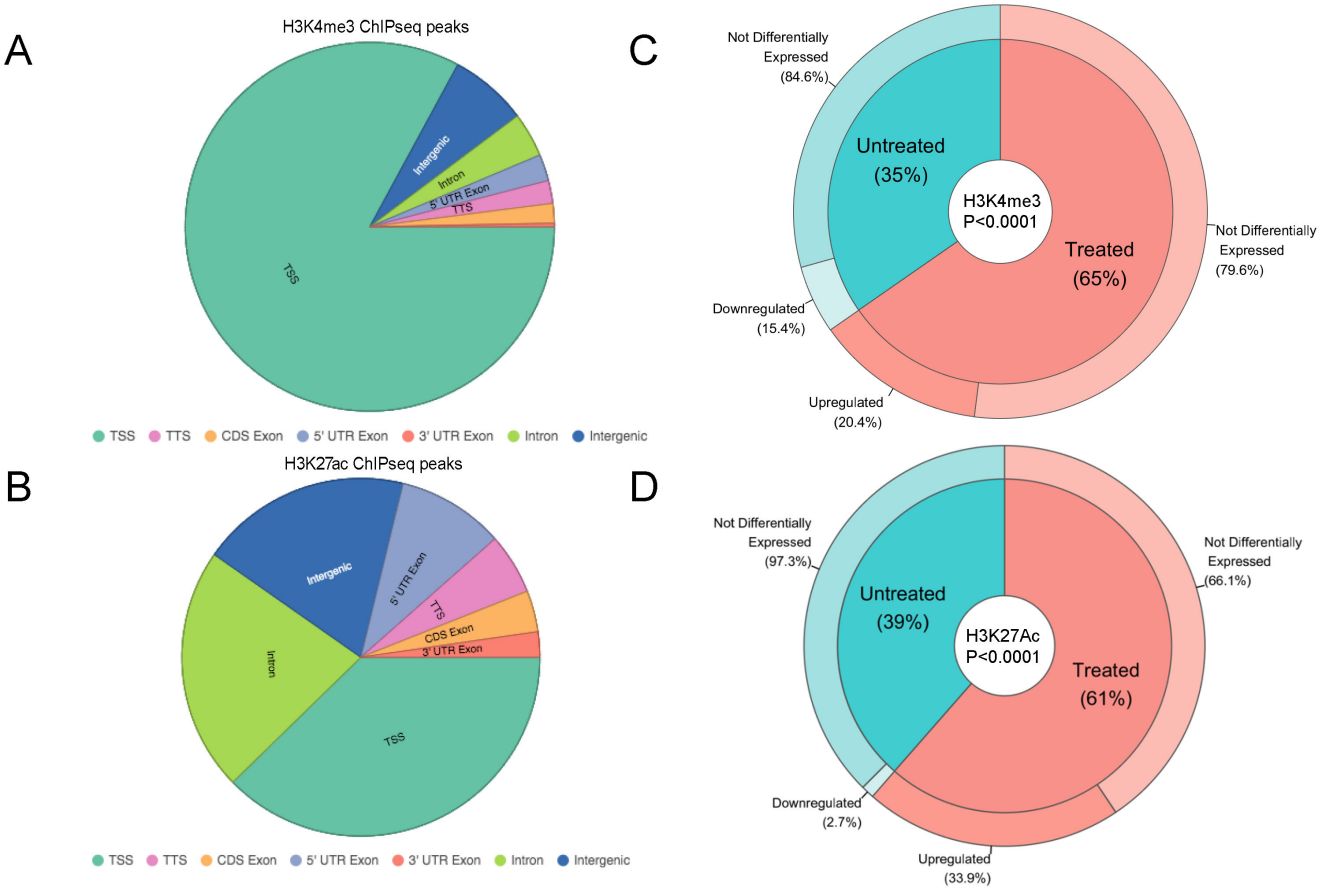
H3K4me3 and H3K27ac marks versus differential gene expression. **A, B**, Pie charts displaying the distribution of H3K4me3 **(A)**, or H3K27ac ChIPseq peaks **(B)** in the LiNKT cells of αGalCer/CD1d-NP-treated and control NOD mice (data pooled for both treatment groups; no differences were noted between the two groups). **(C)** Donut pie representing H3K4me3 peaks found in LiNKTs from control (inner blue pie slice) vs. αGalCer/CD1d-NP-treated mice (inner red pie slice). The relationship between H3K4me3 deposition and gene expression is represented in the outer layers of the donut. Whereas 20.4% of genes with treatment induced H3K4me3 deposition were upregulated, none were downregulated (P = 0.0004). Likewise, whereas 15.4% of the genes that lost H3K4me3 in response to treatment were downregulated, none were upregulated (P = 0.0187). Data were analyzed with Chi-square test. **(D)** Donut pie representing H3K27ac peaks found in LiNKTs from control (inner blue pie slice) vs. αGalCer/CD1d-NP-treated mice (inner red pie slice). The relationship between H3K27ac deposition and gene expression is represented in the outer layers of the donut. Whereas 33.9% of genes with treatment induced H3K27ac deposition were upregulated, none were downregulated (P<0.0001). Likewise, whereas 2.7% of the genes that lost H3K27ac in response to treatment were downregulated, none were upregulated (P<0.0001). Data were analyzed with Chi-square test. Data correspond to 1-1.5x10^6^ LiNKT cells/treatment group and histone modification type, isolated from n=6 control NOD mice and n=6 αGalCer/CD1d-NP-treated NOD mice.

We mapped the location of these two histone marks in the LiNKT cells of αGalCer/CD1d-NP-treated and control NOD mice via chromatin immunoprecipitation followed by sequencing (ChIPseq). We identified a total of 29,310 peaks for H3K4me3. As expected, most of these peaks mapped to TSS (24,302/29,310; 82.91%), followed by intergenic (2,006/29,310; 6.48%) and intronic (1,140/29,310; 3.89%) sites (**Figure 3A**). We also identified a total of 76,616 H3K27ac peaks, most of them near the TSS (28,878/76,616; 37.69%) or at intronic (16,886/76,616; 22.04%) or intergenic (14,607/76,616; 19.07%) sites (**Figure 3B**).

The LiNKT cells of αGalCer/CD1d-NP-treated NOD mice had differentially enriched deposition of H3K4me3 at only 94 sites (FDR≤0.01 vs. the LiNKT cells of control mice) (Datasheet 3). These peaks were associated with 75 genes, 26 and 49 of which had lost (FC<0; 26/75; 35%) or gained (FC>0; 49/75 genes, 65%) H3K4me3 marks in response to treatment (**Figure 3C**), respectively. Analysis of these data in the context of changes in gene expression indicated that whereas 10 of the 49 genes (20.4%) that gained H3K4me3 were upregulated (FC≥4 and FDR≤0.01), including *Il10*, *Lag3* and *Vdr* (**Figure 3C**), none was downregulated (P = 0.0004). A similar percentage of the genes that had lost H3K4me3 (4/26 genes; 15.4%) were downregulated upon treatment (FC ≤ 0 and FDR≤0.01), none being upregulated (**Figure 3C**) (P = 0.0187). Similar results were obtained when we focused these analyses on 46 iNKT-relevant genes (16) (**Supplementary Table 1**). Five of the 13 genes that were upregulated in response to treatment (38.46%), such as *Il10, Lag3, Itga4, Pdcd1* and *Vdr*, but none of the 3 downregulated genes, had enriched deposition of H3K4me3 (P=0.0976).

Similarly to H3K4me3, the LiNKT cells of αGalCer/CD1d-NP-treated NOD mice had enriched deposition of H3K27ac at only 113 sites on 96 genes (FDR≤0.01 vs. the LiNKT cells of control mice) (Datasheet 4). Whereas 37 of these genes lost H3K27ac (39%; FC<0), 59 genes gained H3K27ac (61%; FC>0) in response to treatment (**Figure 3D**). Interestingly, whereas only 1 of the 37 genes that had lost H3K27ac (*Tnfrsf10b*; 2.7%) was downregulated, 20/59 of the genes that had gained H3K27ac (including *Il10*, *Il21*, *Lag3*, *Nfia*, *Ctla4* and *Vdr*; 33.9%) were upregulated (P<0.0001).

These data indicate that treatment-induced upregulation of LiNKT cell gene expression is largely dissociated from *de novo* changes in H3K4me3 or H3K27ac marks, events that only took place in a very small number of genes.

### Gene upregulation vs. treatment induced H3K27ac and H3K4me3 co-deposition and chromatin exposure

About half of the few genes that were both upregulated and differentially marked with H3K4me3 in response to treatment, were also differentially marked with H3K27ac (5/10 genes), including *Il10*, *Lag3* and *Vdr* (Datasheet 5). Furthermore, some of the genes that were differentially marked with H3K4me3 (15/75; 20%) and/or H3K27ac (28/96; 29.16%) in response to treatment, including the LiNKTR1 genes *Il10, Lag3*, *Pdcd1 and Nfia*, acquired new OCRs in response to treatment (Datasheet 6). Thus, LiNKTR1 cell formation in response to αGalCer/CD1d-NP treatment does involve limited epigenetic remodelling of certain LiNKTR1 genes. However, at the global level, treatment induced LiNKT gene upregulation is largely dissociated from the *de novo* appearance of OCRs or H3K4me3/H3K27ac marks.

### Acquisition of new active enhancers is not a critical step in LiNKT to LiNKTR1 reprogramming

Whereas ATACseq helps identify areas of open chromatin associated with various regulatory elements such as enhancers, silencers, and promoters, H3K27ac ChIPseq helps locate class I active enhancer and promoter elements (36). To identify active enhancers in LiNKT and αGalCer/CD1d-NP-induced LiNKTR1 cells, we carried out an integrated analysis of both data sets. Open chromatin regions containing H3K27ac marks, excluding those located within 2 kb of transcription start sites (TSS) (i.e., overlapping promoters), were considered to represent active enhancers. A 100 kb window around the TSS was used to annotate genes proximal to the identified active enhancers (Datasheet 7).

We identified 47,169 active enhancers in the LiNKT cells of untreated mice. Of these, 32,390 (68.67%; linked to 17,604 genes) were located in gene bodies, and 14,779 (31.33%; linked to 8,454 genes) were located in intergenic regions. There were 51,840 active enhancers in αGalCer/CD1d-NP-induced LiNKT cells, most located in gene bodies (35,668 enhancers in 18,924 genes; 68.80%) and in intergenic locations (16,172 enhancers in 9,487 genes; 31.19%). Importantly, most of the genes with active enhancers at the treatment-induced LiNKT cell stage already had these active enhancers at the pre-treatment LiNKT cell stage (gene body: 16,066/18,924, 84.9%; intergenic: 7,448/9,497, 78.42%) (Datasheet 7).

To investigate a potential role for treatment-induced activation of enhancers in treatment-induced LiNKT cell re-programming, we investigated if gene upregulation in response to treatment was accompanied by the presence of associated active enhancers. In agreement with the above data, 100 of the 162 genes that were upregulated by LiNKT cells in response to treatment (61.73%) already had at least one active enhancer in the gene body before the initiation of treatment. Likewise, 48 of the 76 genes that were downregulated by treatment (63.12%) also had active enhancers in their gene bodies at the pre-treatment LiNKT cell stage (P=0.4160). With regards to intergenic active enhancers, they were also already present in 56 of the 162 genes that were subsequently upregulated by treatment (34.57%) and in 17 of the 76 genes that were downregulated by treatment (22.37%) (P=0.0571).

**Figure 4:**
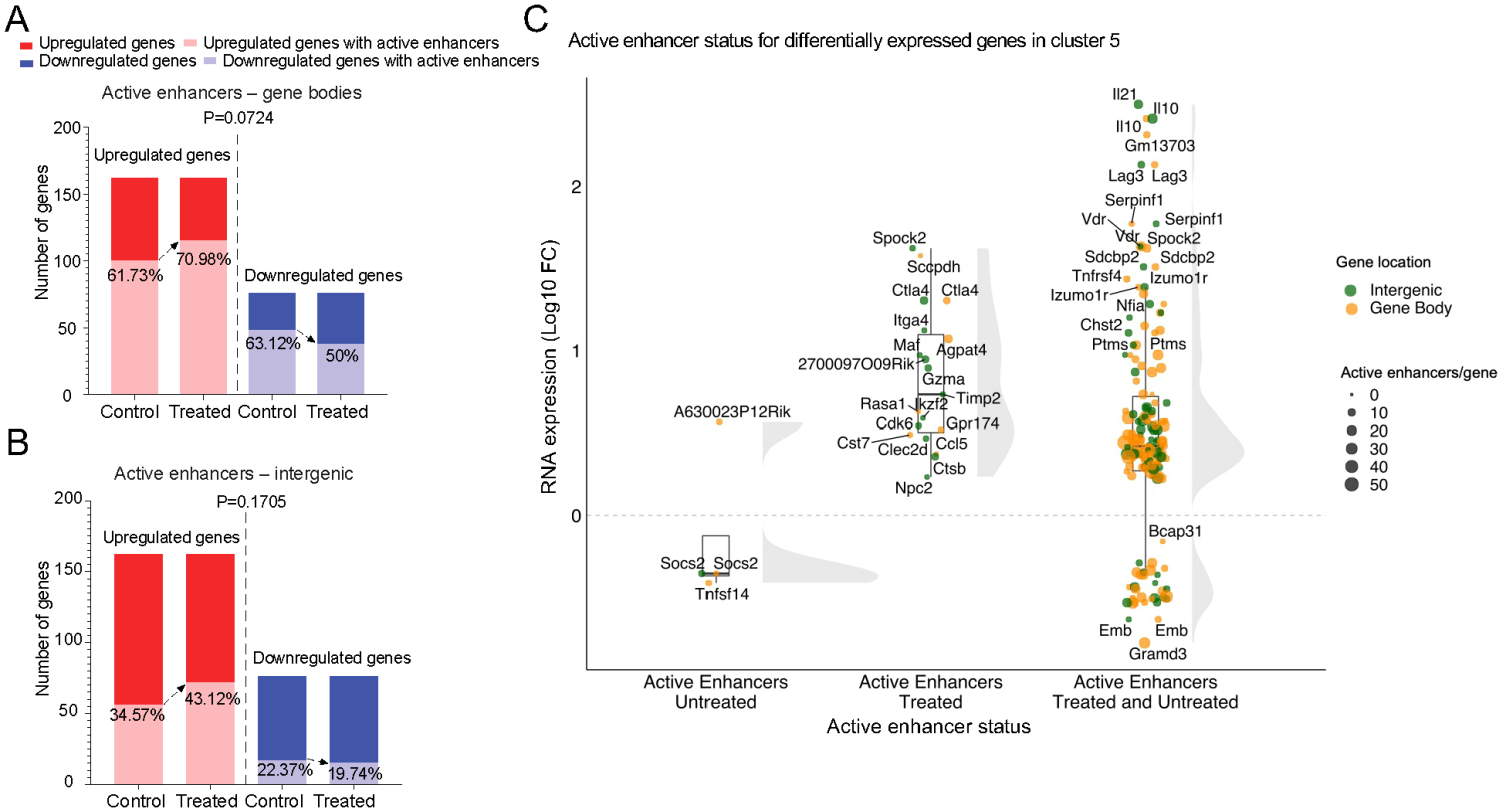
Gene upregulation vs. treatment-induced active enhancers. **(A)** Bar plots depicting the number and percentage of genes harboring active enhancers in the body of genes that are upregulated (left bars; light red) or downregulated (right bars; light blue) in response to αGalCer/CD1d-NP treatment. **(B)** same as in A, but for genes harboring active enhancers in intergenic regions. P values in A and B were >0.05 (Chi-square test). **(C)** Raincloud plots of expression changes (log10FC expression changes from bulk RNAseq data) for genes that are differentially expressed in the αGalCer/CD1d-NP-induced LiNKTR1 sub-cluster (16), as a function of whether they carry active enhancers only in control mice (untreated; left), only in treated mice (treated; middle), or in both (untreated and treated; right). Most of the genes that acquire active enhancers during the LiNKT-to-LiNKTR1 conversion are upregulated (middle group), as compared to genes that lose active enhancers in response to treatment (left group) (P<0.0001). The largest changes in gene expression (upregulation) are seen in genes that already possess active enhancers at the pre-treatment LiNKT cell stage (P=0.0007). Data analyzed with Chi-square test.

Although treatment slightly increased the presence of active enhancers in the gene bodies of upregulated genes (from 100 genes before treatment to 115 genes after treatment; 61.73% to 70.98%, respectively) and triggered the loss of active enhancers in the gene bodies of downregulated genes (from 48 genes before treatment to 38 genes after treatment; 63.12 to 50%, respectively), these differences were not statistically significant (P=0.0724 for presence of active enhancers in upregulated vs downregulated genes) (**Figure 4A**). This was also true when focusing on intergenic active enhancers. Treatment increased the number of genes having intergenic enhancers (from 56 to 72; 34.57% to 43.12%) and decreased the number of downregulated genes having these enhancers (from 17 to 15; 22.37 to 19.74%) (P=0.1705 for lack of active enhancers in downregulated vs upregulated genes) (**Figure 4B**).

To further investigate a potential relationship between presence/lack of active enhancers and differential gene expression during the LiNKT-to-LiNKTR1 differentiation process, we focused on the genes that were specifically upregulated or downregulated in the treatment-induced LiNKTR1 subset. Specifically, we compared the levels of expression of these genes, as a function of whether they had active enhancers in the LiNKT cells of both untreated and treated mice (i.e., genes poised for upregulation at the LiNKT cell stage), only in the LiNKT cells of treated mice (i.e., induced by treatment), or only in the LiNKT cells of untreated mice (i.e., genes in which treatment erases pre-existing active enhancers, or does not elicit the *de novo* appearance of active enhancers). The raincloud plot in **Figure 4C** shows that most of the genes that acquire active enhancers during the LiNKT-to-LiNKTR1 conversion are upregulated (middle group), as compared to genes that lose active enhancers in response to treatment (left group) (P<0.0001). However, the largest changes in gene expression (upregulation) are seen in genes that already possess active enhancers at the pre-treatment LiNKT cell stage (P=0.0007).

Taken together, and in keeping with our earlier observations de-coupling upregulation of the expression for most genes from *de novo* appearance of treatment-induced OCRs or H3K4me3/H3K27ac marks, these data suggest that the genes that are upregulated in response to αGalCer/CD1d-NP treatment already appear to be epigenetically poised to do so at the precursor LiNKT cell stage.

### Treatment-induced iNKT gene upregulation is associated with gene de-methylation

We next performed genome-wide bisulfite sequencing of LiNKT cell samples from αGalCer/CD1d-NP-treated and untreated NOD mice to study the potential role of gene hypo- or hyper-methylation in differential gene expression. We focused our analysis on differentially methylated regions (DMRs) between these samples. With a q-value threshold of 0.05, we detected 2,873 DMRs (Datasheet 8). We associated these DMRs (either hyper- or hypo-methylated in LiNKT cells from treated vs. untreated mice) to gene bodies (intragenic CpG islands) and gene promoters (within 2 kb from the TSS) or intergenic regions (Datasheets 9, 10).

In αGalCer/CD1d-NP-induced LiNKT cells, there were 3,701 genes that contained at least one differentially hypo-methylated region in the gene body (as compared to LiNKT cells from untreated controls), 237 genes that contained differentially hypo-methylated promoters, and 1,514 genes that contained differentially hypo-methylated intergenic regions (Datasheet 11).

**Figure 5:**
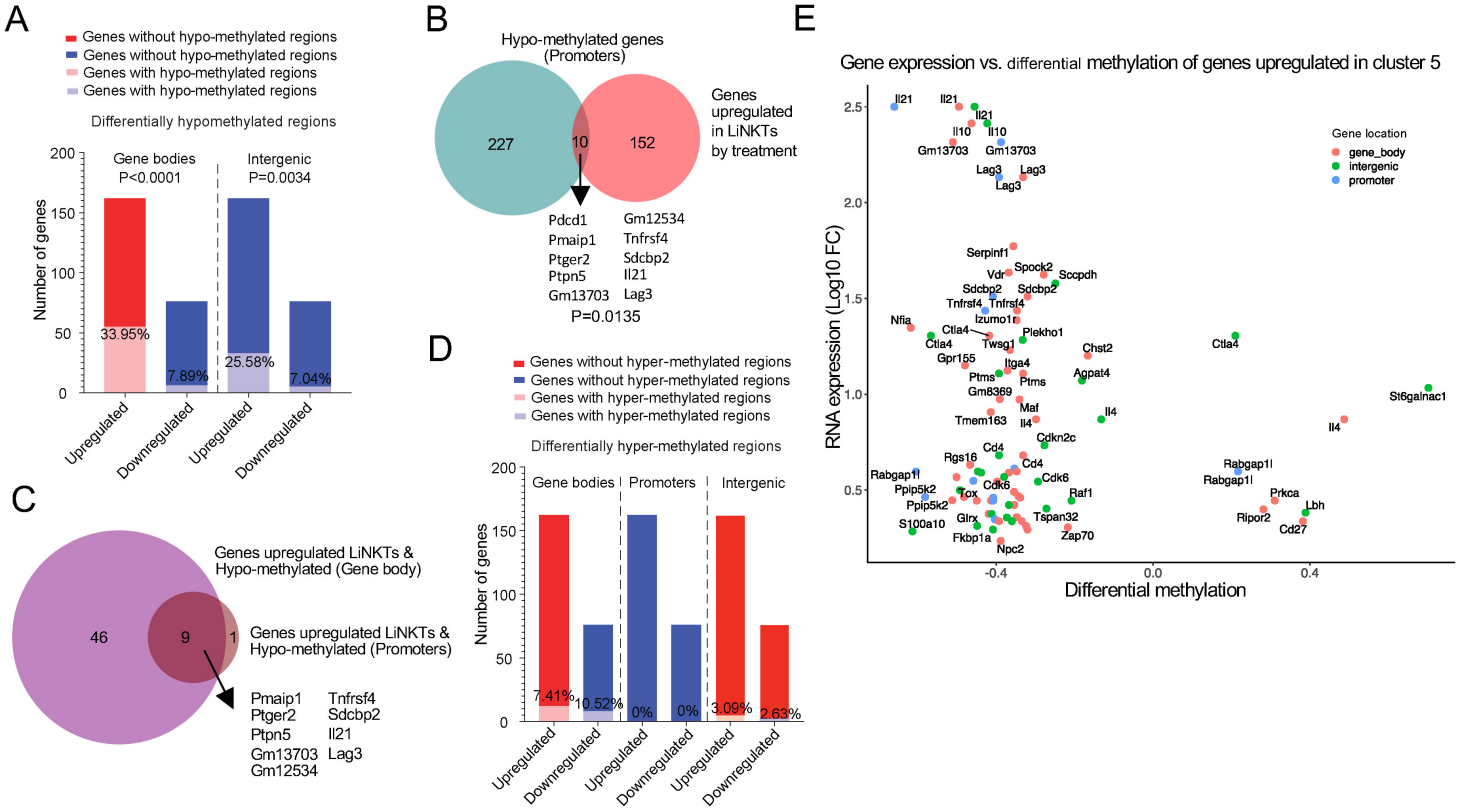
Treatment-induced gene hypo- or hyper-methylation vs. differential gene expression. **(A)** Bar plots depicting the number and percentage of the genes upregulated (red) or downregulated by treatment (blue) that acquired at least one differentially hypo-methylated region in the gene body (left) or in intergenic regions (right) in response to treatment. There was a statistically significant correlation between treatment-induced hypo-methylation in gene bodies (P<0.0001) and intergenic regions (P=0.0034) and gene upregulation (Chi-square test). **(B)** Venn diagram linking treatment-induced de-methylation of promoters with gene upregulation (P=0.0135; Chi-square test). **(C)** Venn diagram linking treatment-induced de-methylation in both gene bodies and promoters with gene upregulation. **(D)** Same as in A, but for treatment-induced hyper-methylation. Red, upregulated genes; blue, downregulated genes. None of the differences were statistically significant (Chi-square test). **(E)** Scatter plot comparing the differential methylation log2 fold change (Log2FC) (“x” axis) of genes specifically upregulated in the treatment induced LiNKTR1 sub-cluster versus the corresponding Log2FC of gene expression (“y” axis). Data correspond to 3 replicates of LiNKTs per treatment group (4-5x10^5^ cells/sample), n=7-17wk old NOD mice and n=4 αGalCer/CD1d-NP-treated 16 wk-old NOD mice).

There was a significant relationship between hypo-methylation, particularly within genes and at intergenic regions but also in promoters, and differential gene upregulation (FC≥4 and FDR≤0.01) in treatment-induced vs. pre-treatment LiNKT cells. For example, 33.95% of the genes upregulated by treatment-induced LiNKT cells (55/162 genes) contained at least one differentially hypo-methylated region within the gene body, as compared to only 7.89% of the downregulated genes (6/76 genes) (**Figure 5A**, left bars) (P<0.0001). This association also held true when focusing on the iNKT-relevant genes listed in **Supplementary Table 1**; ~92% of the upregulated genes (n=12/13) had hypo-methylated gene bodies (*Ctla4, Il4*, *Il10*, *Il21, Itga4, Izumo1r, Lag3, Maf, Nfia, Tigit, Tnfrsf4* and *Vdr*), as compared to only ~33% of the iNKT-relevant genes that were downregulated in response to treatment, which were: *Ccr5, Ccr9, Cd44, Ifng, Irf4, Nfil3, Prdm1, Tbx21, Tox, Zbtb16, Zfp683* (11/33) (P=0.0002) (Datasheet 12). Likewise, ~20% of the genes upregulated by treatment-induced LiNKT cells (33/162), but only ~7% of the downregulated genes (5/76) were associated with hypo-methylated regions located within a 100 kb window around the gene (**Figure 5A**, right bars) (P=0.0034).

αGalCer/CD1d-NP-induced LiNKTR1 cell formation was also accompanied by promoter hypo-methylation, albeit to a lesser extent than that seen in gene bodies and intergenic regions. Specifically, ~6% of the genes that are upregulated in treatment-induced vs. pre-treatment LiNKT cells (10/162), including *Il21, Pdcd1* and *Lag3* contained hypo-methylated promoters (**Figure 5B**), as opposed to none of the 76 downregulated genes (0/76; P=0.0135). Similar associations between promoter hypo-methylation and gene expression were observed when focusing on the iNKT relevant genes from **Supplementary Table 1**; ~31% of the upregulated genes (4/13; *Il21*, *Lag3*, *Pdcd1* and *Tnfrsf4*) but only ~9% of their downregulated counterparts (3/33; *Ccr5*, *Ifng* and *Il2rb*) contained hypo-methylated promoters (P=0.0327). In total, 56 of the 162 genes that were upregulated in response to treatment carried a hypo-methylated gene body or promoter, and 9 of these genes (16.07%), including *Il21* and *Lag3*, were hypo-methylated at both locations (**Figure 5C**).

αGalCer/CD1d-NP-induced LiNKTR1 cell formation was also accompanied by gene hyper-methylation. There were 2,334 genes that contained at least one differentially hyper-methylated region in the gene body (as compared to LiNKT cells from untreated controls), 135 genes that contained differentially hyper-methylated promoters, and 512 genes that contained differentially hyper-methylated intergenic regions (Datasheet 11). In this case, however, neither gene upregulation nor downregulation in response to treatment was statistically associated with differential hyper-methylation in gene bodies (12/162 vs. 8/76 genes, respectively), promoters (0/162 and 0/76, respectively), or intergenic regions (5/162 vs 2/76, respectively) (**Figure 5D**).

Collectively, the above data indicates that αGalCer/CD1d-NP treatment and LiNKTR1 cell formation is accompanied by both DNA hypo- and hyper-methylation, but only the former is associated with changes in gene expression, specifically gene upregulation. Indeed, a significant percentage of the genes that are upregulated by LiNKT cells in response to treatment (LiNKTR1-specific) carry differentially hypo-methylated gene bodies, inter-genic regions and/or promoters (**Figure 5E**).

### Treatment-induced LiNKT-to-LiNKTR1 cell differentiation is positively associated with de-methylation of distal gene regulatory elements (GREs)

As noted above, a significant number of the differentially hypo-methylated regions that arise in response to treatment lie in intergenic regions, possibly distal GREs (Datasheet 11). Several lines of evidence suggest that patterned hypo-methylation signatures at GREs might reflect stable markers of cell identity. DNA methylation generally inhibits transcription (37), even at enhancers and enhancer de-methylation is cell type-specific and predicts target gene transcription (38). In addition, differential methylation among cell types is greatest at distal GREs, rather than promoters (39, 40), which is also the case for αGalCer/CD1d-NP-induced LiNKTR1 cells (see above). Furthermore, de-methylation at these sites is usually a required final step in activation of enhancers and stabilization of cell line identities (41).

**Figure 6:**
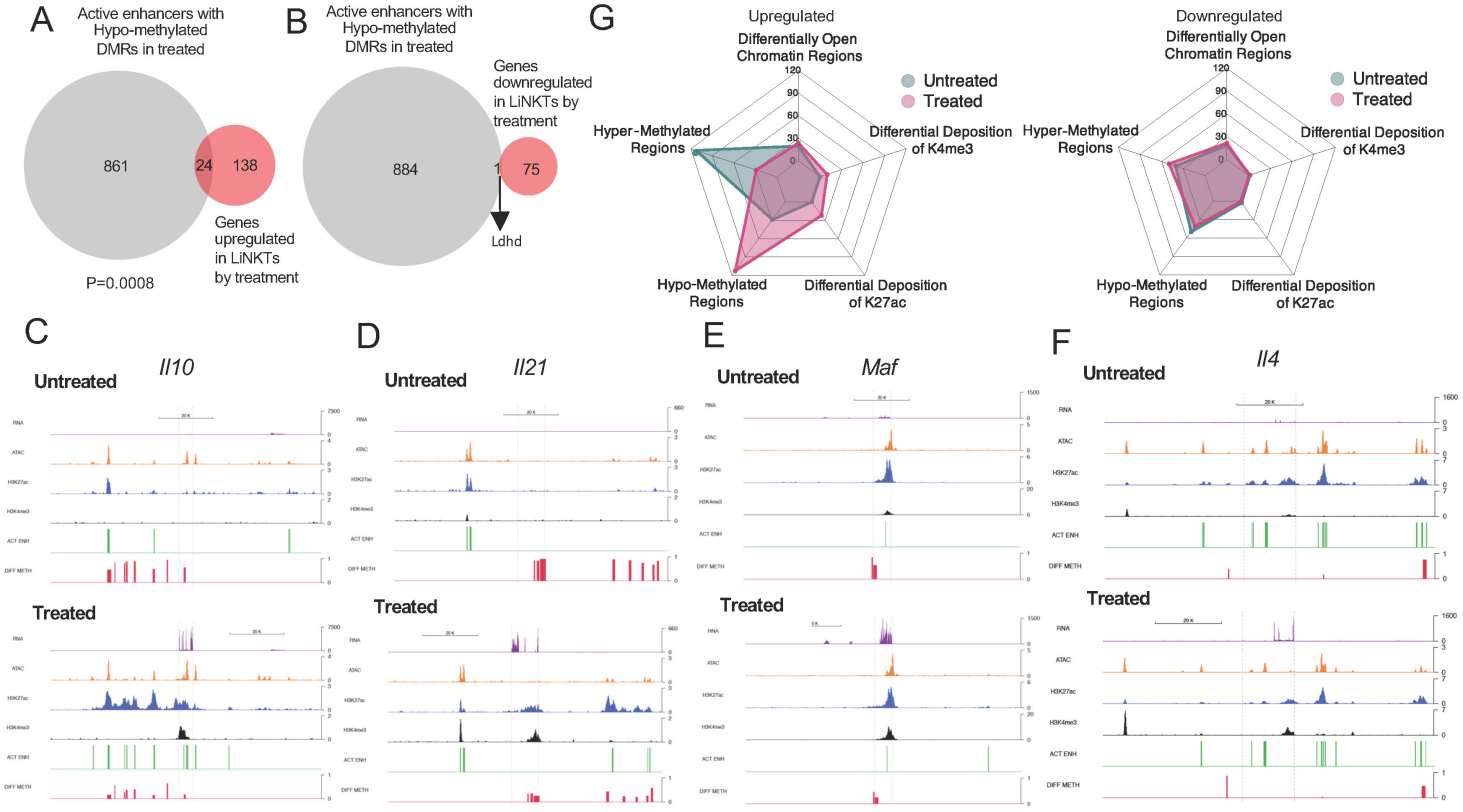
Relative contribution of different epigenetic marks to changes in gene expression during the LiNKT to LiNKTR1 cell conversion. **(A, B)**, Venn diagrams linking treatment-induced de-methylation of active enhancers and gene upregulation **(A)** or downregulation **(B)**. There was a statistically significant correlation between de-methyation and gene upregulation (P=0.0008; Chi-square test). **(C-F)** RNA tracks (purple) and the location of OCRs (orange), H3K27ac (blue), H3K4me3 (black), active enhancers (green) and DMRs (red) for representative Group #1-4 genes (*Il10*, *Il21*, *Maf* and *Il4*, respectively). **(G)** Radar plots depicting the weight of each epigenetic mark based on the number of significant occurrences, on treatment-induced (in sub-cluster 5) gene upregulation (left) or downregulation (right). Note the major effect of treatment-induced DNA hypo-methylation on gene upregulation.

We thus investigated how many of the active enhancers found in αGalCer/CD1d-NP-induced LiNKTR1 cells colocalize with DMRs and to what extent hypo-methylation of these enhancers might contribute to gene upregulation. 840/51,840 (1.62%) of the active enhancers found in αGalCer/CD1d-NP-induced LiNKTR1 cells had undergone hypo-methylation during the LiNKT to LiNKTR1 cell transition. These 840 hypo-methylated active enhancers were proximal (within 50 kb upstream or downstream of the TSS) to 885 genes. Whereas 24 of these 885 genes (2.71%; *Gm43822, Rgs8, Rgs16, Gm37019, Gzmk, Runx2os1, Lgmn, Il4, Esm1, Gm12534, Alcam, Gzma, Nfia, F730043M19Rik, Angptl2, Nipal1, Ptger2, Ctla4, Il10, Gpr155, A430093F15Rik, Gm13703, Itga4, Il21*) were significantly upregulated in response to treatment (FC≥4 and FDR≤0.01) (**Figure 6A**), only 1 gene (0.11%; *Ldhd*) was downregulated (FC≤4 and FDR≤0.01) (**Figure 6B**) (P=0.0008) (Datasheets 13, 14).

Thus, treatment-induced LiNKT-to-LiNKTR1 cell differentiation is, to certain extent, positively associated with de-methylation of distal GREs, consistent with a cell re-programming event (39, 40).

### Relative contribution of different epigenetic marks to changes in gene expression during the LiNKT to LiNKTR1 cell conversion

To define the relative contribution of the various epigenetic modifications discussed above on gene expression, we focused on the 13 iNKT-relevant genes from **Supplementary Table 1** that are specifically upregulated in response to αGalCer/CD1d-NP treatment. Four of these genes (*Il10*, *Lag3, Ctla4* and *Nfia*) contained differential OCRs and H3K27ac marks and were hypo-methylated (Group 1). Three other genes (Group 2: *Il21*, *Tnfrsf4* and *Vdr*) did not contain differential OCRs but were differentially marked with H3K27ac and were hypo-methylated. In three additional genes (Group 3: *Maf*, *Itga4* and *Pdcd1*), the treatment induced gene hypo-methylation and the acquisition of new OCRs but not new H3K27ac marks. The remaining three genes *(*Group 4: *Il4, Tigit* and *Izumo1r*) underwent hypo-methylation in response to treatment but did not acquire new OCRs or H3K27ac marks. **Figures 6C-F** depicts the RNA tracks and the location of H3K27ac and H3K4me3 marks, OCRs, active enhancers and DMRs for representative Group #1-4 genes (*Il10*, *Il21*, *Maf* and *Il4*, respectively). Thus, all the 13 iNKT-relevant genes that were upregulated in response to treatment had undergone hypo-methylation, and in 10 of these genes hypo-methylation was accompanied by other epigenetic changes.

**Figure 7:**
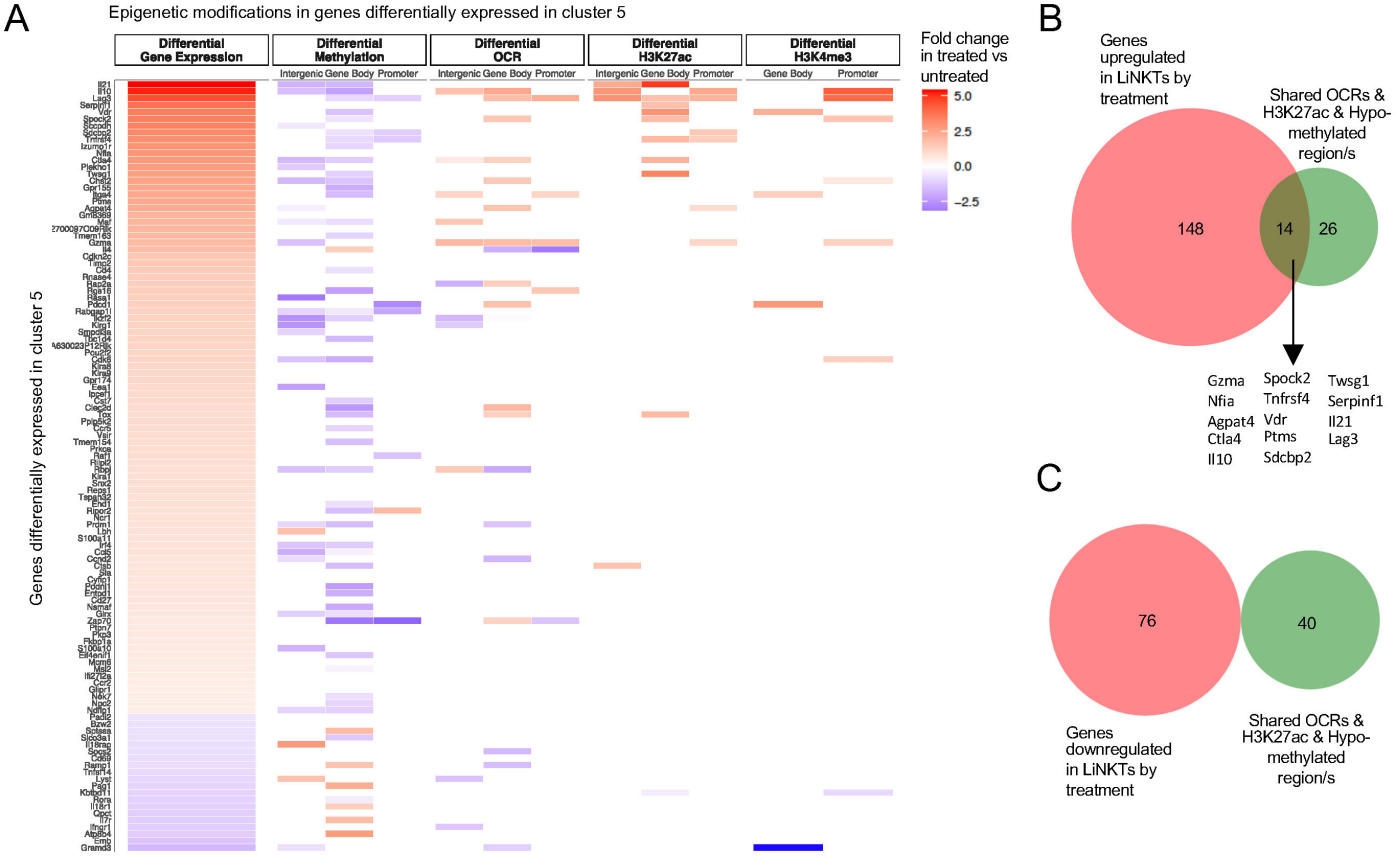
The role of different epigenetic marks in differential expression of LiNKTR1 (sub-cluster 5)-specific genes that shared at least one OCR with the LiNKT cells from untreated mice. **(A)** Heatmap depicting the presence of different epigenetic marks (from left to right: differential methylation, differential OCRs, differential H3K27ac deposition and differential H3K4me3 deposition) in LiNKTR1 genes arranged from most to least upregulated. **B, C**, Venn diagrams linking treatment-induced gene upregulation **(B)** or downregulation **(C)** for genes sharing at least one OCR with pre-treatment LiNKT cells, with treatment-induced H3K27ac deposition gains and DNA hypo-methylation (all regions considered for hypo-methylation) (P<0.0001 for upregulation vs downregulation; Chi-square test).

To further explore the relative weight of the various epigenetic modifications studied herein on gene expression, we extended the above studies to all the genes that are specifically expressed/differentially upregulated by the treatment induced LiNKTR1 cell cluster, which is absent in untreated mice. The radar plots shown in **Figure 6G** suggest that treatment induced hypo-methylation plays a major role in differential gene upregulation. A similar outcome was obtained when focusing on the LiNKTR1-specific genes that shared at least one OCR with the LiNKT cells from untreated mice (**Figure 7A**). Whereas for a significant number of differentially expressed genes, gene upregulation is largely associated with treatment-induced hypo-methylation, the most upregulated genes (i.e. *Il10* and *Il21*, among others) are also those that accumulate additional epigenetic modifications favoring gene expression, such as new OCRs and H3K27ac and H3K4me3 marks. Of the 40 genes that had undergone hypo-methylation and increased H3K27ac deposition in response to treatment, and shared least one or more OCRs with pre-treatment LiNKT cells, 14 (35%; including *Il10*, *Il21*, *Ctla4*, *Lag3*, *Nfia* and *Vdr*) were upregulated (**Figure 7B**), and none had undergone downregulation (P<0.0001) (**Figure 7C**) (Datasheet 15).

These observations thus suggest that the changes in gene expression that accompany the LiNKT-LiNKTR1 transdifferentiation process in response to repetitive and sustained ligation of TCRs largely involve expression-promoting changes in DNA methylation that are generally not accompanied by other epigenetic modifications. Exceptions to this de-methylation-only pattern include LiNKTR1 lineage-defining genes such as *Il10* and *Lag3*, where there is also increased chromatin accessibility and enrichment for H3K27ac and H3K4me3 deposition, possibly suggesting the contribution of putative super-enhancers in this process.

### Epigenetic stability of LiNKT versus TFH and Th0 cells

Through a combination of bulk and single-cell transcriptional and epigenetic studies, we have recently shown that TFH cells and pMHCII-NP-induced TR1-like cells (arising from cognate TFH precursors) are epigenetically very similar to each other, yet remarkably different from their Th0 precursors, where changes in gene expression are accompanied by major changes in chromatin structure, transcription-enhancing histone deposition maps, and de-methylation of gene bodies and gene regulatory sequences (24). The data described herein on LiNKT-to-LiNKTR1 cell re-programming, involving significant changes in the LiNKT methylome in the absence of other epigenetic modifications, is in part reminiscent of the epigenetic stability of TFH cells as they transdifferentiate into TR1 cells (24).

**Figure 8:**
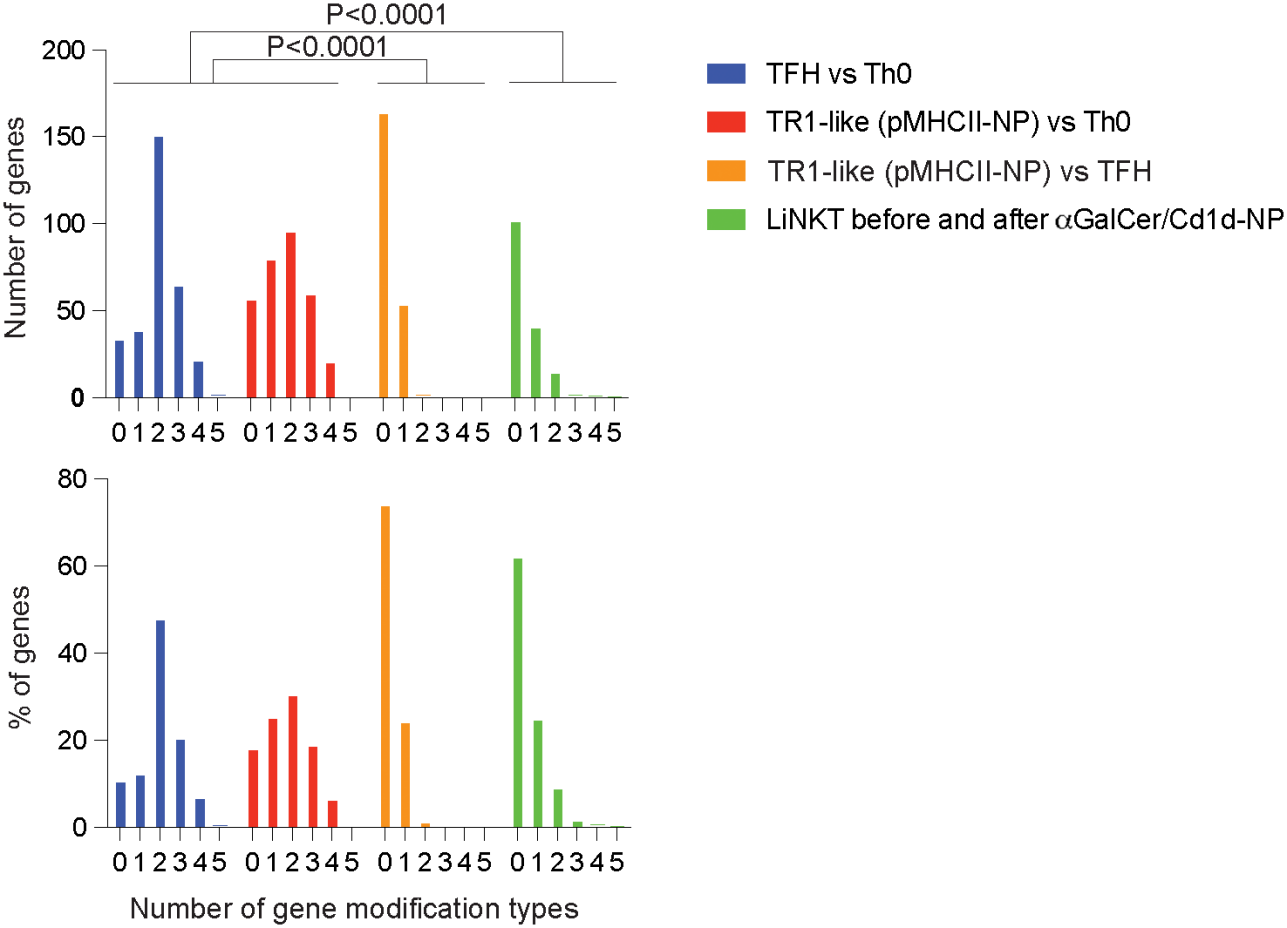
Scope of epigenetic modifications underpinning the re-programming of LiNKT cells in response to αGalCer/CD1d-NPs as compared to the Th0-TFH and TFH-TR1 differentiation pathways. Number of significant gene-associated epigenetic modification types (y-axis; DNA methylation, chromatin accessibility, and H3K27ac and H3K4me3 deposition) among the genes that are differentially expressed in αGalCer/CD1d tetramer+ LiNKT cell clusters 1-4 upon repetitive αGalCer/CD1d-NP encounters, as compared to Th0 vs antigen-induced TFH, pMHCII-NP-induced TR1-like vs Th0 or antigen-induced TFH vs. pMHCII-NP-induced TR1-like cells (24). Top shows absolute number of genes; bottom corresponds to % of genes. Data were compared via Chi-square.

We thus compared the extent with which splenic TFH cells or LiNKT cells changed their epigenomes in response to MHC-NP challenge. As shown in **Figure 8** and **Supplementary Figure 2**, most of the TR1 and LiNKTR1 cell genes that were differentially upregulated or downregulated in response to MHC-NP treatment acquired fewer than 2 epigenetic modifications (among the 5 investigated). However, whereas changes in gene expression in LiNKT cells upon αGalCer/CD1d-NP challenge were significantly associated with changes in gene methylation, changes in gene expression in TFH cells upon pMHCII-NP challenge were not (24). In contrast, most of the TFH cell genes that were differentially expressed versus Th0 cells had acquired at least 2 or more modification types. Thus, LiNKT cells, though resistant to most of the epigenetic modifications that Th0 cells undergo to become TFH cells, are particularly susceptible to changes in DNA methylation affecting gene expression.

## Discussion

The work described herein sought to investigate the epigenetic underpinnings of the αGalCer/CD1d-NP-induced reprogramming of LiNKT cells into immunoregulatory LiNKTR1 progeny (16). Although our data suggest that LiNKT cells are relatively resistant to global epigenetic reprogramming in response to sustained TCR ligation *in vivo*, they indicate that such cells are particularly susceptible to changes in DNA methylation affecting gene expression.

TCRαβ sequencing indicated that LiNKTR1 cell generation in response to αGalcer/CD1d-NP engagement is neither preceded, nor accompanied by significant LiNKT cell proliferation, supporting a direct conversion of LiNKTs into LiNKTR1 cells. This is in contrast to what has been reported for a single i.p or i.v. injection of soluble αGalCer, which is accompanied by adipose tissue and LiNKT proliferation as measured by Ki67 expression, bromodeoxyuridine (BrdU) incorporation and/or cell cycle gene expression analysis (42). Likewise, these results are at odds with the pMHCII-NP-induced transdifferentiation of cognate TFH cells into TR1 CD4+ T cells, which is immediately preceded by a proliferative burst of antigen-specific TFH cells (22). Thus, whereas αGalCer/CD1d-NP-induced signaling into LiNKT cells triggers the expression of a TR1-like transcriptional program, this event is not preceded by proliferation of LiNKT or LiNKTR1 cells.

In keeping with the peripheral heterogeneity of tissue-resident iNKT cells (1, 43, 44), the αGalCer/CD1d-reactive LiNKT cell pool comprises 4 different cell subclusters. As shown previously, αGalCer/CD1d-NP treatment triggers the *de novo* appearance of a fifth subcluster that expresses a TR1-like transcriptional program (16). The single cell multiome analyses of LiNKT cells from untreated and treated mice reported herein suggest that the LiNKTR1 subcluster (cluster #5) arises from subclusters 1 and/or 2, based on similar open chromatin landscapes and transcriptional make-up. Indeed, our scATACseq data revealed that the treatment-induced upregulation or downregulation of gene expression in LiNKT cells leading to LiNKTR1 cell formation are largely dissociated from the *de novo* appearance or closure of OCRs. Although our epigenetic studies of the LiNKT-LiNKTR1 cell conversion have focused on NOD mouse LiNKT cells, our previous work has demonstrated that αGalCer/CD1d-NP-induced reprogramming of LiNKT cells into LiNKTR1 progeny is not a unique property of the NOD genetic background; it also occurs in both C57BL/6 and NOD.*c3c4* mice (16).

Importantly, although treatment-induced upregulation of LiNKT cell gene expression is positively associated with increased deposition of H3K4me3 and H3K27ac marks at promoters or distal gene regulatory elements of certain genes, it is largely dissociated from the *de novo* appearance of H3K4me3/H3K27ac marks. Furthermore, although acquisition and loss of active enhancers by LiNKT cells in response to treatment do play a modulatory effect in gene expression, the acquisition of new active enhancers does not appear to be a critical step in treatment-induced LiNKT cell reprogramming.

These observations contrast with the important roles that H3K4me3 and H3K27ac deposition play in T helper cell differentiation. For example, upregulation of lineage-specific genes in Th1 cells, such as *Ifng*, is associated with TCR signaling-induced/STAT-dependent deposition of expression-enhancing H3K27ac and H3K4me3 marks on promoters (45), and removal of repressive marks (26). In fact, removal of the latter at bivalent enhancers and promoters of key Th1/Th2-specific cytokine and transcription factor genes, such as *Tbx21* and *Gata3*, are thought to play a major role in T-helper cell differentiation (25–27).

The presence of methyl groups on cytosine-guanine dinucleotide pairs (CpGs) sterically inhibit transcription factor positioning and promote the recruitment of inhibitors of gene expression that recognize DNA methylation. It is noteworthy that the DNA demethylases TET2 and TET3 have been shown to play a critical role in *Tbx21* (T-bet), *Il2rb* (IL2RB) or *Zbtb7b* (ZBTB7B) expression by iNKT cells; in their absence, iNKT cells downregulate IFN-γ expression and shift towards an iNKT17-like phenotype (46, 47). Likewise, the ubiquitin-like containing PHD and RING finger domain-1 (UHRF1) factor, which recruits the DNMT1 DNA methyltransferase has been shown to play an essential role in thymic iNKT cell development (47, 48). Related to this, here we find that αGalCer/CD1d-NP treatment-induced gene upregulation, is positively associated with *de novo* DNA hypo-methylation of gene bodies, promoters and/or distal regulatory elements. For example, the *Zbtb16* gene, coding for the iNKT transcription factor PLZF, undergoes significant de-methylation as LiNKT cells upregulate PLZF expression and transdifferentiate into LiNKTR1 cells. PLZF regulates the expression of genes encoding various LiNKT and LiNKTR1 cytokine receptors, adhesion and homing molecules, by binding to TFs such as GATA-3, c-MAF or RORγ, and by repressing *Bach2* (49). In fact, when we compare the relative weight of the various epigenetic modifications studied herein on gene expression, we find that treatment induced hypo-methylation plays the most significant role. However, the most upregulated genes (i.e. the regulatory cytokines *Il10* and *Il21*, and the co-inhibitory molecules *Lag3* and *Ctla4*) are those that accumulate additional epigenetic modifications favoring gene expression, such as new OCRs and H3K27ac and H3K4me3 marks. Since DNA de-methylation can have a positive effect on H3K27ac, and H3K4me3 deposition can inhibit the recruitment of DNA methyltransferases and thus suppress DNA silencing deposition (50), it is possible that these changes are coordinately regulated to help stabilize the expression of key LiNKTR1 genes. In most cases, however, changes in DNA methylation during the LiNKT-to-LiNKTR1 conversion are generally not accompanied by other epigenetic modifications.

Changes in DNA methylation are also known to play a significant role in the expression of certain T helper cell subset-specific genes. For example, during Th2 differentiation, *Il4*, *Il5* and *Il13*, which are hyper-methylated in naïve T cells (28), are de-methylated and acquire various expression-enhancing histone marks (29, 30). Likewise, *Tbx21*, encoding the Th1 transcription factor T-bet, becomes hyper-methylated in Th2 cells (31). Some of these changes are mediated, at least in part, by the Th2 transcription factor GATA-3 (51, 52). In LiNKTR1 cells, αGalCer/CD1d-NPs upregulated the expression of *Il4* without increasing the expression of *Gata3*, which in fact gained hyper-methylated regions in the gene body. Another example of de-methylation-associated gene upregulation in T cells is *Foxp3*. Whereas in naïve T cells, the *Foxp3* promoter is hyper-methylated, it becomes de-methylated (and marked with H3K27ac and H3K4me3) in FoxP3+ Treg cells (32), either in response to TCR and/or IL-2 and TGF-β signaling (33, 53).

It could be argued that studies modulating the expression/function of Ten-eleven translocation (TET) family proteins and DNA methyltransferases in iNKT cells might provide additional insights into the role of gene de-methylation on the LiNKT-to-LiNKTR1 conversion. However, these studies face significant technical challenges. The αGalCer/CD1d-NP-induced LiNKT-to-LiNKTR1 differentiation pathway, similar to the pMHCII-NP-induced TFH-to-TR1 conversion (24), is a context-dependent *in vivo* process that cannot be reproduced *in vitro*. Systemic *in vivo* modulation of DNA methylation may not be informative, as it would affect physiological processes confounding data interpretation. Our current work seeks to address these limitations by defining the identity of the LiNKT cell subset that gives rise to LiNKTR1 progeny, using conditional transcription factor knock-out mice. Selective modulation of DNA methylation in such cells would overcome, to a significant extent, these caveats.

The above observations confirm that LiNKTs, like their splenic counterparts (15) can indeed remodel their epigenome in response to potent antigenic stimulation, in particular through changes in DNA methylation. However, comparison of the epigenetic events underpinning the differentiation of LiNKT cells into LiNKTR1 cells with those associated with the naïve T cell–TFH and TFH–TR1 differentiation pathways reveal significant differences and similarities, respectively. When compared to Th0 cells, TFH and TR1-like cells possess remarkably different epigenetic landscapes, consistent with the epigenetic plasticity of naive CD4+ T cells, where changes in gene expression leading to TFH generation, for example, are accompanied by major changes in chromatin structure, transcription-enhancing histone deposition maps, and de-methylation of gene bodies and gene regulatory sequences. In contrast, antigen-induced TFH and pMHCII-NP-induced TR1-like cells (arising from cognate TFH precursors) are epigenetically very similar to each other (24). Thus, like TFH cells, but unlike Th0 cells, LiNKT cells are resistant to undergoing large epigenetic modifications of their genome.

In sum, the work reported herein sheds light on the epigenetic mechanisms underlying the differentiation of LiNKT cells into regulatory LiNKTR1 cells, highlighting a dominant role for DNA demethylation. This finding has significant implications for our understanding of iNKT cell biology: it implies that one or more subsets of LiNKT cells (i.e., those giving rise to the LiNKTR1 subset) exhibit unique epigenetic plasticity, largely associated with changes in gene methylation, and that such plasticity allows them to transition into regulatory LiNKT cells. This exposes LiNKT cells as *bona fide* targets for the treatment of autoimmune liver inflammation or other liver inflammatory processes, such as, for example, allogeneic liver transplant rejection. LiNKT cell-targeted modulation of the key epigenetic events underpinning the LiNKT-to-LiNKTR1 differentiation process might help promote this process. For example, by selectively modulating DNA methylation pathways, it might be possible to enhance the therapeutic efficacy of LiNKT cell-based treatments, such as the approach described herein.

## Methods

### Mice

Male and female non-obese diabetic NOD/ShiLtj mice (strain #001976) were obtained from The Jackson Laboratory (Bar Harbor, ME, USA) and their official distributor Charles River Laboratory (Wilmington, MA, USA). Mice were maintained in a specific-pathogen free (SPF) environment with free access to water and food. All experiments detailed within this study were approved by the Cumming School of Medicine of the University of Calgary animal care committee and by the University of Barcelona’s animal ethics committee.

### αGalCer/Cd1d monomer and tetramer production

Empty mouse CD1d monomers were purified from supernatants of CHO-S cells (Invitrogen, San Diego, CA) transduced with lentiviruses encoding a monocistronic message in which β2m and CD1d were separated by the ribosome skipping P2A sequence. The CD1d monomers were engineered to encode a BirA site, a 6×His tag and a free Cys at the carboxyterminal end of the construct. The self-assembled CD1d complexes were purified by nickel chromatography. The purified mCD1d monomers were used for coating onto NPs and/or processed for biotinylation and tetramer formation. Briefly, CD1d monomers were biotinylated using a BirA5000 biotin ligase kit (Avidity, Aurora, CO, USA) according to manufacturer’s indications. Biotinylated monomers were subsequently purified using Pierce Monomeric Avidin Kit (Thermo Fisher Scientific, Waltham, MA, USA) and buffer exchanged on PD-10 desalting columns (GE Healthcare, Chicago, IL, USA). Purified biotinylated monomers were loaded with KRN 7000 lipid (Cayman Chemical Company, Ann Harbor, MI, USA) in the presence of 0.05% PBST for 3 hours at 37°C, and incubated with Streptavidin-APC conjugate (Agilent Technologies, Santa Clara, CA, USA) for 16 hours at 4°C to obtain tetramers.

### Nanoparticle synthesis, mCD1d conjugation and αGalCer loading

Maleimide-functionalized, pegylated iron oxide NPs (PFM series) were produced in a single-step thermal decomposition in the absence of surfactants as described (17). Briefly, 3g Maleimide-PEG (2 kDa MW, Jenkem Tech USA) were melted in a 50mL round bottom flask at 100°C and then mixed with 7 mL of benzyl ether and 2mmol Fe(acac)_3_. The reaction was stirred for 1 hr and heated to 260°C with reflux for 2 hr. The mixture was cooled to room temperature and mixed with 30 mL water. Insoluble materials were removed by centrifugation at 2,000xg for 30 min. The NPs were purified using magnetic (MACS) columns (Miltenyi Biotec, Auburn, CA) and stored in water at room temperature or 4°C. The concentration of iron was determined spectrophotometrically at 410 nm in 2N hydrochloric acid (HCl). To conjugate mCD1d onto PFM, mCD1d monomers were mixed with NPs in 40 mM phosphate buffer, pH 6.0, containing 2mM ethylenediaminetetraacetic acid (EDTA), 150mM NaCl, and incubated overnight at room temperature. mCD1d-conjugated NPs were purified by magnetic separation and concentrated by ultrafiltration through Amicon Ultra-15 (100 kDa cut-off) and stored in PBS. To load αGalCer onto mCD1d-NPs, KRN 7000, dissolved in DMSO, was added to the mCD1d-NP suspension at a molar ratio of 12:1, respectively in the presence of 0.05% PBST and incubated for 3 h at 37°C followed by overnight at 4°C. The αGalCer-loaded CD1d-NPs were subjected to magnetic purification and then sterilized by filtration through 0.2μm filters and stored at 4°C. The size and dispersity of unconjugated and mCD1d-conjugated NPs were assessed via transmission electron microscopy (TEM, Hitachi H7650) and dynamic light scattering (DLS, Zetasizer, Malvern, UK). Pegylated and CD1d/pMHC-NPs were also analyzed via 0.8% agarose gel electrophoresis and native- and denaturing 10% SDS-PAGE.

### αGalCer/CD1d-NP treatment

Cohorts of male and female NOD mice aged 8-10 weeks were intravenously (i.v.) injected with αGalCer/CD1d-NPs in PBS at 20 µg protein/dose in a final volume of 200 μl twice a week for 5 weeks using Ultra-Fine™ 30G insulin syringes (BD, Franklin Lakes, NJ, USA). Control mice were left untreated or treated with Cys-NPs (the exact same amount of iron given with the αGalCer/CD1d-NPs).

### Liver iNKT cell staining and sorting

Mice were bled to completion by severing the heart and abdominal aortas. Liver cell suspensions were subjected to 37.5% isotonic Percoll gradient (Percoll, Sigma-Aldrich) centrifugation in the presence of heparin (10 U/ml), to separate red blood cells (RBCs) and immune cells from non-immune liver cells. Upon hemolysis of RBCs using a red blood cell lysis solution (Miltenyi Biotec), single cell suspensions were sequentially stained with anti-mouse CD16/CD32 mAb for 15 min at room temperature (clone 93; BioLegend), followed by αGalCer/CD1d tetramer (3 to 5 μg/ml, at room temperature for 1h) and anti-mouse TCRβ FITC or PE mAb (5 µg/ml) for 30 min at 4°C (clone H57-597; BD Biosciences). Live TCRβ^int^ tetramer^+^ cells were sorted using FACSAria II, FACSAriaIII or FACSAria SORP instruments (BD Biosciences, Franklin Lakes, NJ, USA). Dead cells were excluded by staining with 7-ADD Viability dye from BD biosciences. BD sorter files were analyzed using FlowJo software (BD). Mouse iNKT cells were isolated 2-3 days after the last αGalCer/CD1d-NP dose.

### 10X single-cell RNA-seq

Alive cells were collected from untreated and αGalCer/CD1d-NP-treated 13-15 wk-old NOD mice in DMEM media (Sigma-Aldrich) supplemented with 10% FBS (Hyclone) at 4 °C, separated into nanoscale gel beads emulsions with a 10X barcode. Cell numbers and viability were assessed using a TC20™ Automated Cell Counter (Bio-Rad Laboratories, Hercules, CA, USA), with a minimum target of 4000 cells. Later, cDNA sequencing libraries were produced using the NextGEM Single-cell 3’ mRNA kit (v3.1; 10X Genomics) following the manufacturer’s instructions. These steps involved GEM-RT clean-up, cDNA Amplification for 13 cycles, and cDNA quality control and quantification using the Agilent Bioanalyzer High Sensitivity chip (Agilent Technologies). Libraries were indexed by PCR using the PN-220103 Chromium i7 Sample Index Plate. Finally, sequencing was carried out on a NovaSeq 6000 sequencer (Illumina).

### 10X single-cell multiome (scRNAseq+scATACseq)

For 10X multiome RNA-seq+ATAC-seq, LiNKT cells were sorted from untreated (n=5; 13-15wk-old) and αGalCer/CD1d-NP-treated male NOD mice (n=4; 13-15wk-old), to obtain a total of 300.000 and 250.000 live LiNKT cells in DMEM media (Sigma-Aldrich) supplemented with 10% FBS (Hyclone). Cells were lysed at 4°C, and nuclei isolated. Nuclei were used for transposition of adapter sequences and processed for single-cell barcoding and library generation following the manufacturer’s instructions (CG000338; 10X Genomics). Briefly, isolated nuclei were partitioned into Gel Bead-In-Emulsions to produce barcoded cDNA from poly-adenylated mRNA as described above, as well as barcoded DNA fragments, and processed for library amplification and sequencing on a NovaSeq 6000 sequencer (Illumina), also as described above.

### Smartseq2 scRNAseq

One-cell sorting of LiNKTs from untreated and αGalCer/CD1d-NP-treated mice (n=3 male mice/group; 13-15 wk-old) was performed in SMARTseq2 96-well plates (PerkinElmer, Waltham, MA, USA) containing 100 µl lysis buffer/well, consisting in 0.2% v/v Triton-100 with RNase inhibitor. Full-length single-cell RNA sequencing libraries were prepared using a modified Smart-seq protocol (54). A reverse transcription reaction was done in presence of oligo-dT30VN, betaine and template-switching oligonucleotides. Complementary DNA was then amplified with KAPA Hifi Hotsart RadyMix (Kappa Biosystems) and ISPCR primer in 25 cycles. The PCR product was purified with Agencourt Ampure XP beads (Beckman Coulter) and analyzed with a High Sensitivity DNA kit (Agilent Technologies). 200 pg of the cDNA product were fragmented with Nextera^®^ XT (Illumina) and amplified with indexed Nextera^®^ PCR primers. Products were purified twice with Agencourt AMPure XP beads and quantified again using a Bioanalyzer High Sensitivity DNA Kit. Sequencing of Nextera^®^ libraries from 384 cells was carried out using one sequencing lane on an Illumina HiSeq2500 v4 or HiSeq4000 to 500K reads/cell.

### ATACseq

ATAC-seq was done on LiNKT cells isolated from untreated and αGalCer/CD1d-NP-treated mice (n=3 male mice/group; 13-15 wk-old). 7x10^4^ cells were sorted in PBS and processed for library preparation as described (55). Briefly, cells were lysed in cold lysis buffer (10 mM Tris-HCl, pH 7.4,10 mM NaCl, 3 mM MgCl2, and 0.1% IGEPAL CA-630) to isolate nuclei. The purified nuclei were washed, resuspended in the transposase reaction mix (25 μl 2x TD buffer, 2.5 μl transposase (Illumina) and 22.5 μl nuclease-free water) and incubated for 30 min at 37°C. DNA was purified with MinElute Reaction Cleanup kit (Qiagen). Libraries were generated with NEBNext^®^ High Fidelity PCR kit (New England Biolabs, Ipswich, MA, USA) using 1x NEB Next PCR master mix (New England BioLabs) and 1.25 μM of custom Nextera PCR primers. Libraries were rendered using the barcoded primers Ad1_noMX as forward and Ad2.1-6 as reverse and purified using a PCR cleanup kit (Qiagen), yielding a final concentration of about 30 nM in 20 μl. Libraries were then analyzed on Bioanalyzer using an Agilent DNA High Sensitivity chip (Agilent Technologies, Santa Clara, CA, USA) to estimate the quantity and size distribution. Next, they were quantified by qPCR using the KAPA Library Quantification Kit before amplification with Illumina’s cBot. Libraries were finally loaded at 3.33 pM onto the flowcell and sequenced 1 x 50 on Illumina’s HiSeq 2500 to obtain 30-40 million reads/sample.

### ChIP-seq

Chromatin immunoprecipitation (ChIP) and sequencing was performed on LiNKT cell samples from untreated and αGalCer/CD1d-NP-treated NOD mice (n=3/group; 13-15 wk-old) for H3K4me3and H3K27Ac bound DNA via ChIP-seq following van Arensbergen’s protocols (56). LiNKT cells were sorted, fixed with 16% paraformaldehyde (PFA; Electron Microscopy Sciences, Hatfield, PA, USA) in DMEM (Sigma-Aldrich) supplemented with 10% FBS (Hyclone) and frozen at -80°C until processed. We used 1-1.5x10^6^ LiNKTs/sample. Cells were lysed, sheared, and sonicated using an S220 Focused-ultrasonicator (Covaris, Woburn, MA, USA) (13 min, 105 W, 2% Duty Factor, 200 cycles). This was followed by overnight incubation with the precipitating antibody: 0.5 µL of H3K4me3 (Sigma), and 2 µL of H3K27Ac (Abcam, Cambridge, United Carlsbad, CA, USA) and precipitated using Protein-A-Dynabeads (Abcam). RNA was cleared using RNAse A (Qiagen) (1 hour at 65°C), and decrosslinking was performed overnight with proteinase K at 65°C. DNA was finally purified with Phenol-Chloroform and EtOH-precipitation. After quality control validation on a Bioanalyzer (Agilent Technologies), samples were sent for sequencing. Libraries were prepared using the NEB Next Ultra DNA Library Prep kit (Illumina) following the manufacturer’s protocol, analyzed with DNA High Sensitivity kit (Agilent Technologies), and quantified via qPCR using the KAPA Library Quantification Kit (Kapa Biosystems). Libraries were loaded at a concentration of 2.75 pM onto flowcells and were sequenced 1 x 50 on Illumina’s HiSeq 2500 to get 30-40 million reads/sample.

### Methylome

Six replicates of LiNKTs (4-5x10^5^ cells/sample) were obtained from untreated female NOD mice (n=7, 17wk-old) and αGalCer/CD1d-NP-treated NOD mice (n=4, 16wk-old) by cell sorting. Genomic DNA was extracted using the DNeasy Blood and Tissue kit (Qiagen) following the manufacturer’s instructions, and frozen at -20°C. Samples were sent to Beijing Genomics Institute (BGI, Shenzhen, China) for sequencing. DNA was processed by Whole Genome Bisulfite Sequencing (WGBS). Briefly, DNA was sonicated to a mean size of 250 bp using a Bioruptor (Diagenode, Seraing (Ougrée), Belgium) followed by blunt-ending, dA addition to the 3’-end and ligation of methylated adaptors to protect from bisulfite conversion. Ligated DNA was bisulfite converted using the EZ DNA Methylation-Gold kit (ZYMO, Irvine, CA, USA). After treatment with sodium bisulfite, unmethylated cytosine residues were converted to uracil, leaving 5-methylcytosine (5mC) unaffected. Different insert size fragments were excised from the same lane of a 2% TAE agarose gel. Products were purified using a QIAquick Gel Extraction kit (Qiagen) and amplified by PCR; after PCR amplification, uracil residues were converted to thymine. Sequencing was performed 2 x 150 bp using the NovaSeq 6000 System (Illumina).

### Bioinformatic and statistical analyses

All fastq files obtained for each omics analysis were assessed for quality control metrics before further analysis with the FastQC tool. Sources for the indicated bioinformatic packages and tools are described further below.

#### ATAC-seq and ChIP-seq

For bulk ATAC-seq analysis, Illumina adapters and low-quality bases were first removed from fastq files reads using Trimmomatic. Next, reads were aligned to the GRCm38 mouse genome using bowtie2, and duplicates were removed using Picard’s `MarkDuplicates`. Then peaks were called using MACS2 with a q-value cutoff of 0.05, read extension of 5’->3’ of 150, and keeping duplicates as they had been removed previously (`-q 0.05 –nomodel –extsize 150 –keep-dup all`). The resulting BAM files were processed into bigwig format for genomic track representation using samtools, deeptools, and trackViewer.

For ATAC-seq analysis, which included various replicates, differential open chromatin regions between samples were analyzed using DiffBind using BAM and peakset files. For ChIP-seq data, differential peaks between samples were obtained using GSA (Gene Set Analysis) from Partek^®^. Peaks were annotated using `annotatePeak` from the ChIPseeker package, using the UCSC mm10 reference included in org.Mm.eg.db and TxDb.Mmusculus.UCSC.mm10.knownGene R packages.

#### Methylome

Upstream bioinformatic analysis of whole-genome methylome data was performed by the bioinformatics team at BGI. In short, sequencing data was filtered to remove adaptor sequences and low-quality reads from raw reads. Filtered data was mapped to the *Mus musculus* reference genome (mm10) (parameters for paired-end reads: *-v 9 -z 33 -p 8 -n 0 -w 20 -s 16 -f 10 -L 100*) by BSMAP, and duplication reads were removed and mapping results merged for each library. The mapping and bisulfite conversion rates were measured for each sample to check the quality of the alignment. Only uniquely mapped data were used to get methylation data. Methylation level was determined by dividing the number of reads covering each mC by the total reads covering that cytosine. Differentially methylated regions (DMRs) were identified by comparing control and treated samples’ methylomes in areas that contained at least 5 CpG (CHG or CHH) sites with a 2-fold change in methylation level and Fisher’s test P value ≤ 0.05. DMRs between conditions (untreated vs treated) were calculated using the metilene tool. Adjacent DMRs were considered interdependent or joined into one continuous DMR if all the regions were differentially methylated between samples. Genomic tracks for methylome data were represented using the trackViewer R package.

#### SmartSeq2 RNAseq

For each successfully sequenced cell, STAR v2.5.4b was used to align the reads to the mouse reference genome GRCm38, and to obtain gene counts per cell matrices. TCR sequences were reconstructed from Smart-seq data with TraCeR v.0.5.1. We performed the secondary analysis of gene expression using the Seurat R package, where we first discarded poor quality cells based on features counts and mitochondrial and ribosomal content. Then data were normalized, scaled, and dimensionally reduced using PCA (Principal Component Analysis) and either t-SNE (t-distributed stochastic neighbor embedding) or UMAP (Uniform Manifold Approximation and Projection). Finally, cells were clustered using K-means, and visualization and differential analysis were performed.

#### Single-cell Multiome (scRNAseq+scATACseq)

10X multiomic data of simultaneous RNA-seq and ATAC-seq were analyzed using 10x Genomics software Cellranger-ARC. In short, gene expression matrices from gene expression data were obtained by alignment to reference genome (GRCm38) using STAR. UMIs (reads) and cell barcodes were filtered, grouped, and counted. Cells were called and their gene expressed reported in matrices based on RNA content for each cell barcode. Transposase accessibility data were processed to remove adapter sequences and trimmed. Alignment was performed using the BWA-MEM algorithm, using a fixed insert size distribution, and duplicates were removed. Peaks were called across all the cells to maximize the signal and then separated by barcode, obtaining peak-barcode matrices. Subsequently, gene expression and peak matrices were combined and analyzed using Seurat and Signac packages. Data were first filtered for poor-quality cells using features and peaks counts, mitochondrial content, nucleosome signal, and transcription start site (TSS) enrichment. RNA and ATAC data were normalized and scaled using `SCTransform` and `RunTFIDF` functions, respectively. Also, each dataset was dimensionally reduced using PCA for RNA and LSI for ATAC and either t-SNE or UMAP. Simultaneous multidimensional reduction of joint RNA-seq and ATAC-seq data was performed using the weighted-nearest neighbor (WNN) algorithm from Seurat and clustered using the `FindClusters` function and K-means functions.

#### Active enhancer prediction

ATAC-seq and H3K27ac-ChIPseq were used to predict potential active enhancer regions. Using the ‘GenomicRanges’ package in R Studio, all peaks called for ATAC-seq overlapping peaks called for H3K27Ac deposition in the same sample, that were not in a promoter region (2 kb region upstream of TSS), were considered active enhancers. The level of differential methylation in these predicted active enhancers was also measured.

#### Chromosome views

We used the trackViewer R package to combined the information from RNAseq, ATACseq, ChIPseq and methylation data in linear plots representing gene tracks for specific genes. Alignment bam files for RNAseq, ATACseq and ChIPseq were obtained from Partek Flow and then transformed to BigWig (input data type for genomicRanges) format using bamCoverage (deepTools) and samtools tools to be uploaded into R.

#### Software and tools used for bioinformatic analyses

BiocManager (v1.30.16) (https://cran.r-project.org/package=BiocManager%0A%0AbiomaRt (v2.48.3)) (57); Bowtie2 (v2.4.2) (58); BSMAP (v3.0) (59); BWA (v0.0.7) (60); Cellranger (v6.0) (https://support.10xgenomics.com/single-cell-gene-expression/software/pipelines/latest/what-is-cell-ranger); Cellranger-arc (v2.0) (https://support.10xgenomics.com/single-cell-multiome-atac-gex/software/pipelines/latest/what-is-cell-ranger-arc); ChipSeeker (v1.28.3) (61); clusterProfiler (v4.0.5) (62) Deeptools (v3.5.0) (63); Deseq2 (v1.32.0) (64); DiffBind (v3.2.7) (65); FastQC (http://www.bioinformatics.babraham.ac.uk/projects/fastqc/); Gene Ontology (66) (http://geneontology.org); Genomic Ranges (v1.44.0) (67); MACS2 (v2.2.7.1) (68); Monocle3 (v1.0.1) (69); org.Mm.eg.db (v3.13.0) (https://bioconductor.org/packages/release/data/annotation/html/org.Mm.eg.db.html); Picard (v2.25.0) (https://broadinstitute.github.io/picard/); R (v4.1.0), R Core Team (2020). — European Environment Agency, n.d.) (https://www.eea.europa.eu/data-and-maps/indicators/oxygen-consuming-substances-in-rivers/r-development-core-team-2006); Rstudio (v1.4.1103) (RStudio | Open Source & Professional Software for Data Science Teams - RStudio, n.d.) (https://www.rstudio.com/); R- trackViewer Bioconductor package (https://github.com/jianhong/trackViewer); Samtools (70) (http://samtools.sourceforge.net); Seurat (v4.0.3) (71); Signac (v1.3.0) (72); STAR (v2.7.10a) (73); Tidyverse (v1.3.1) (https://www.tidyverse.org); Trackviewer (v1.31.1) (74); Trimmomatic (v.039) (75).

## Data Availability

The datasets presented in this study can be found in online repositories. The names of the repository/repositories and accession number(s) can be found below: GSE168488 (bulk RNA-seq data) and GSE250192 (SmartSeq2 scRNA-seq, ATAC-seq, ChIP-seq, methylome, and single-cell multiome data) (GEO).
