# Supplementary material for: Transcriptional re-programming of liver-resident iNKT cells into T-regulatory type-1-like liver iNKT cells involves extensive gene de-methylation
Source: Front Immunol. 2024 Sep 9;15:1454314. doi: 10.3389/fimmu.2024.1454314 (PMC11416961; doi:10.3389/fimmu.2024.1454314)
Supplement: Supplementary file 1 [file DataSheet1.zip › Montaño_Supplementary_Data.pdf]

Supplementary Table 1. List of selected iNKT-relevant markers

Genes (protein name)	iNKT1[a]	iNKT2[a]	iNKT17[a]	iNKT10[a]	FoxP3+ iNKT[a]	ATR iNKT[a]	Breg-induced iNKT[a]	iNKT-FH[a]
Zbtb16 **(Plzf)**	+/–	+	+	–	+	–	+	?
Tbx21 **(Tbet)**	+	–	–	?	?	?	?	?
Gata3 **(Gata-3)**	+/–	+	+	?	?	?	?	?
Rorc **(Rorγt)**	–	–	+	?	?	?	?	?
Foxp3 **(Foxp3)**	–	–	–	–	+	–	–	?
Maf **(Maf)**	–	–	+	?	?	?	?	?
Nfil3 **(E4bp4)**	–	–	–	+	?	+	–	?
Bcl6 **(Bcl6)**	?	?	?	–	?	?	?	+
IRF4 **(Irf4)**	–	+	+	?	?	?	?	?
Lef1 **(Lef-1)**	+	+	+	+	?	?	?	?
Cd24a **(CD24)**	+/–	+/–	+/–	+	?	?	?	?
Cd44 **(CD44)**	+	+	+	?	?	?	?	?
Klrb1b/c **(NK1.1)**	+	–	+/–	+/–	?	+/–	?	?
Ifng **(IFNγ)**	+	–	–	?	?	?	?	?
Il2 **(IL-2)**	–	–	–	?	?	+	?	?
Il4 **(IL-4)**	+	+	–	?	?	+	?	?
Il17a **(IL-17)**	–	–	+	?	?	?	?	?
Il10 **(IL-10)**	–	–	–	+	?	+	?	?
Il21 **(IL-21)**	–	–	–	?	?	?	?	+
Il2rb **(CD122)**	+	–	–	?	?	?	?	?
Mirlet7 **(Let-7)**	+	?	?	?	?	?	?	?
Zfp683 **(Hobit)**	+	?	?	?	?	?	?	?
Il17rb **(IL-17Rβ)**	–	+	+/–	?	?	?	?	?
Tnfsf11 **(Rankl)**	–	+	+	?	?	?	?	?
Tnfrsf4 **(CD134)**	?	?	?	+	?	?	?	?
Il7r **(IL-7R)**	?	?	?	+	?	?	?	?
Tgfbr2 **(Tgfβr2)**	?	?	+	?	?	?	?	?
Lag3 **(Lag-3)**	?	?	?	?	?	?	?	?
Ctla4 **(Ctla-4)**	?	?	?	+	?	?	?	?
Slamf6 **(Slaf6)**	–	+	–	+	?	?	?	?
Itga4 **(CD49d)**	?	?	–	+	?	?	?	?
Itgae **(CD103)**	?	?	+	–	?	?	?	?
Pdcd1 **(Pd1)**	?	?	?	+	?	?	?	+
Tigit **(Tigit)**	?	?	?	?	?	?	?	?
Ccr6 **(Ccr6)**	–	–	+	?	?	?	?	?
Ccr9 **(Ccr9)**	–	+	–	+	?	?	?	?
Cxcr5 **(Cxcr5)**	–	+	–	?	?	?	?	+
ICOS **(Icos)**	+/–	+	+	+	?	?	?	+
Nrp1 **(Nrp1)**	?	?	?	+	?	?	?	?
Izumo1r **(Fr4)**	?	?	?	+	?	?	?	?
Cd27 **(CD27)**	+	+	–	?	?	?	?	?

[a] From references (–, not expressed; +/–, low; +, expressed) ([1, 2, 3, 4, 5, 6, 7, 8, 9, 10, 11, 12, 13, 14])

1. Doisne JM*, et al.* Skin and peripheral lymph node invariant NKT cells are mainly retinoic acid receptor-related orphan receptor (gamma)t+ and respond preferentially under inflammatory conditions. *J Immunol* **183**, 2142-2149 (2009).

2. Monteiro M*, et al.* Identification of regulatory Foxp3+ invariant NKT cells induced by TGF-beta. *J Immunol* **185**, 2157-2163 (2010).

3. Chang PP*, et al.* Identification of Bcl-6-dependent follicular helper NKT cells that provide cognate help for B cell responses. *Nat Immunol* **13**, 35-43 (2011).

4. King IL*, et al.* Invariant natural killer T cells direct B cell responses to cognate lipid antigen in an IL-21-dependent manner. *Nat Immunol* **13**, 44-50 (2011).

5. Schipper HS*, et al.* Natural killer T cells in adipose tissue prevent insulin resistance. *J Clin Invest* **122**, 3343-3354 (2012).

6. Lee YJ, Holzapfel KL, Zhu J, Jameson SC, Hogquist KA. Steady-state production of IL-4 modulates immunity in mouse strains and is determined by lineage diversity of iNKT cells. *Nat Immunol* **14**, 1146-1154 (2013).

7. Sag D, Krause P, Hedrick CC, Kronenberg M, Wingender G. IL-10-producing NKT10 cells are a distinct regulatory invariant NKT cell subset. *J Clin Invest* **124**, 3725-3740 (2014).

8. Lynch L*, et al.* Regulatory iNKT cells lack expression of the transcription factor PLZF and control the homeostasis of T(reg) cells and macrophages in adipose tissue. *Nat Immunol* **16**, 85-95 (2015).

9. Berga-Bolanos R, Zhu WS, Steinke FC, Xue HH, Sen JM. Cell-autonomous requirement for TCF1 and LEF1 in the development of Natural Killer T cells. *Mol Immunol* **68**, 484-489 (2015).

10. Gapin L. Development of invariant natural killer T cells. *Curr Opin Immunol* **39**, 68-74 (2016).

11. Engel I*, et al.* Innate-like functions of natural killer T cell subsets result from highly divergent gene programs. *Nat Immunol* **17**, 728-739 (2016).

12. Crosby CM, Kronenberg M. Tissue-specific functions of invariant natural killer T cells. *Nat Rev Immunol* **18**, 559-574 (2018).

13. Wang H, Hogquist KA. How Lipid-Specific T Cells Become Effectors: The Differentiation of iNKT Subsets. *Front Immunol* **9**, 1450 (2018).

14. Oleinika K*, et al.* CD1d-dependent immune suppression mediated by regulatory B cells through modulations of iNKT cells. *Nature communications* **9**, 684 (2018).

Suppl. Fig. 1

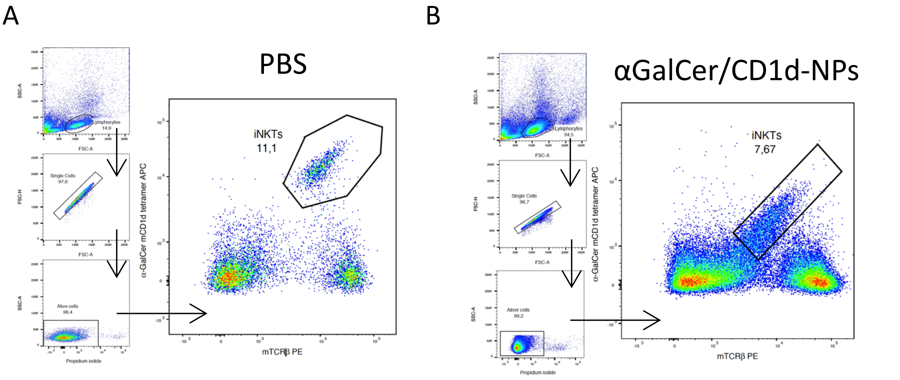
A
B
PBS
αGalCer/CD1d-NPs
Lymphocytes
14,9
Single Cells
97,6
Alive cells
98,4
iNKTs
11,1
FSC-A
SSC-A
FSC-H
Propidium iodide
α-GalCer mCD1d tetramer APC
mTCRβ PE
Lymphocytes
34,5
Single Cells
98,7
Aliver cells
99,2
iNKTs
7,67

Supplementary Fig. 1. Representative LiNKT cell sorting flow cytometry gating profiles. Sorting strategy: Lymphocytes > Single cells > Live cells (propidium iodide–) > LiNKTs (TCRß-FITC^{int}CD1d tetramer-APC+). **A,** Cells from untreated mice. **B,** Cells from mice treated with αGalCer/CD1d-NPs.

A

Suppl. Fig. 2

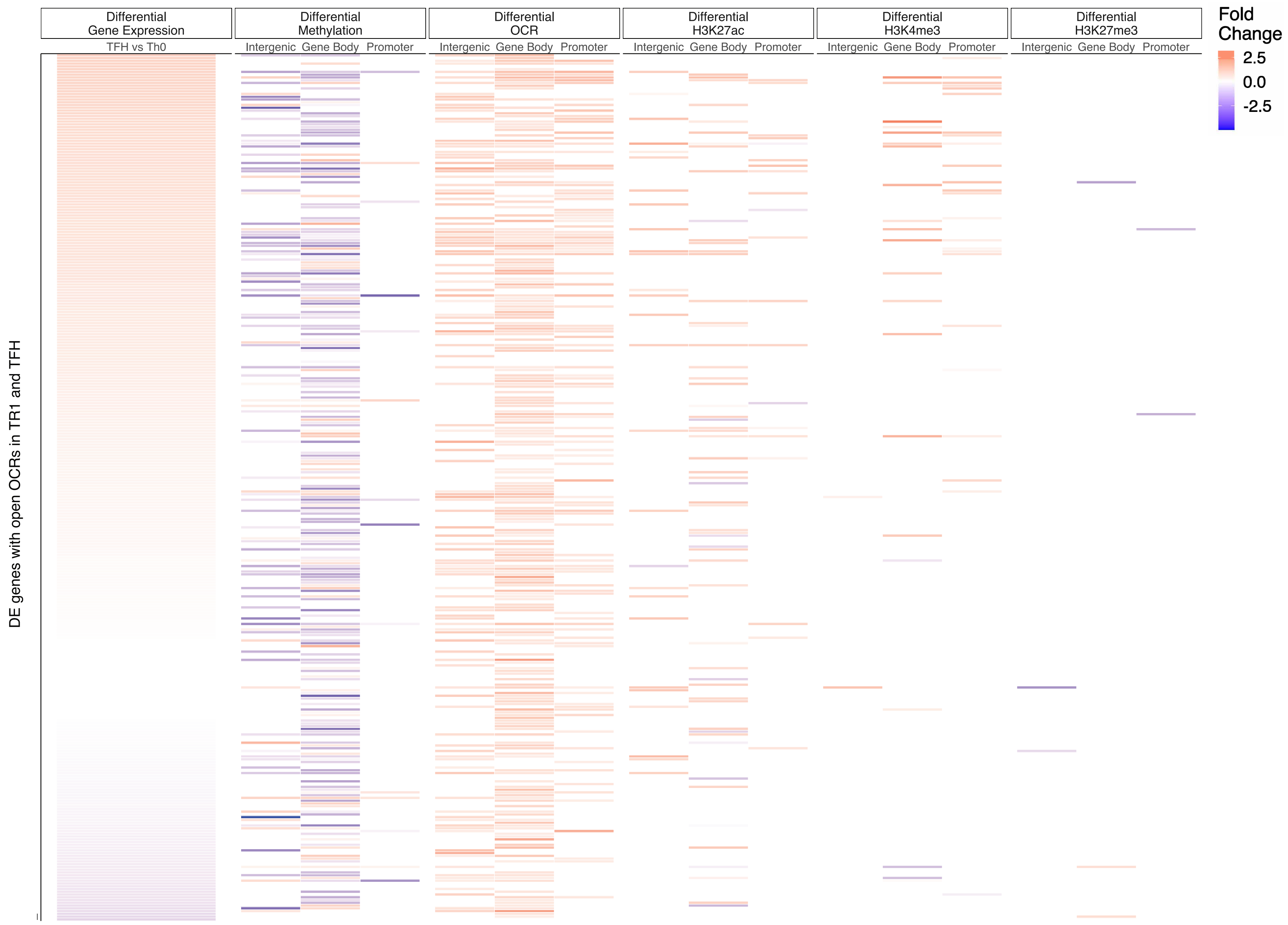

Differential
Gene Expression
TFH vs Th0
Differential
Methylation
Intergenic Gene Body Promoter
Differential
OCR
Intergenic Gene Body Promoter
Differential
H3K27ac
Intergenic Gene Body Promoter
Differential
H3K4me3
Intergenic Gene Body Promoter
Differential
H3K27me3
Intergenic Gene Body Promoter
Fold
Change
2.5
0.0
-2.5
DE genes with open OCRs in TR1 and TFH

B

Suppl. Fig. 2

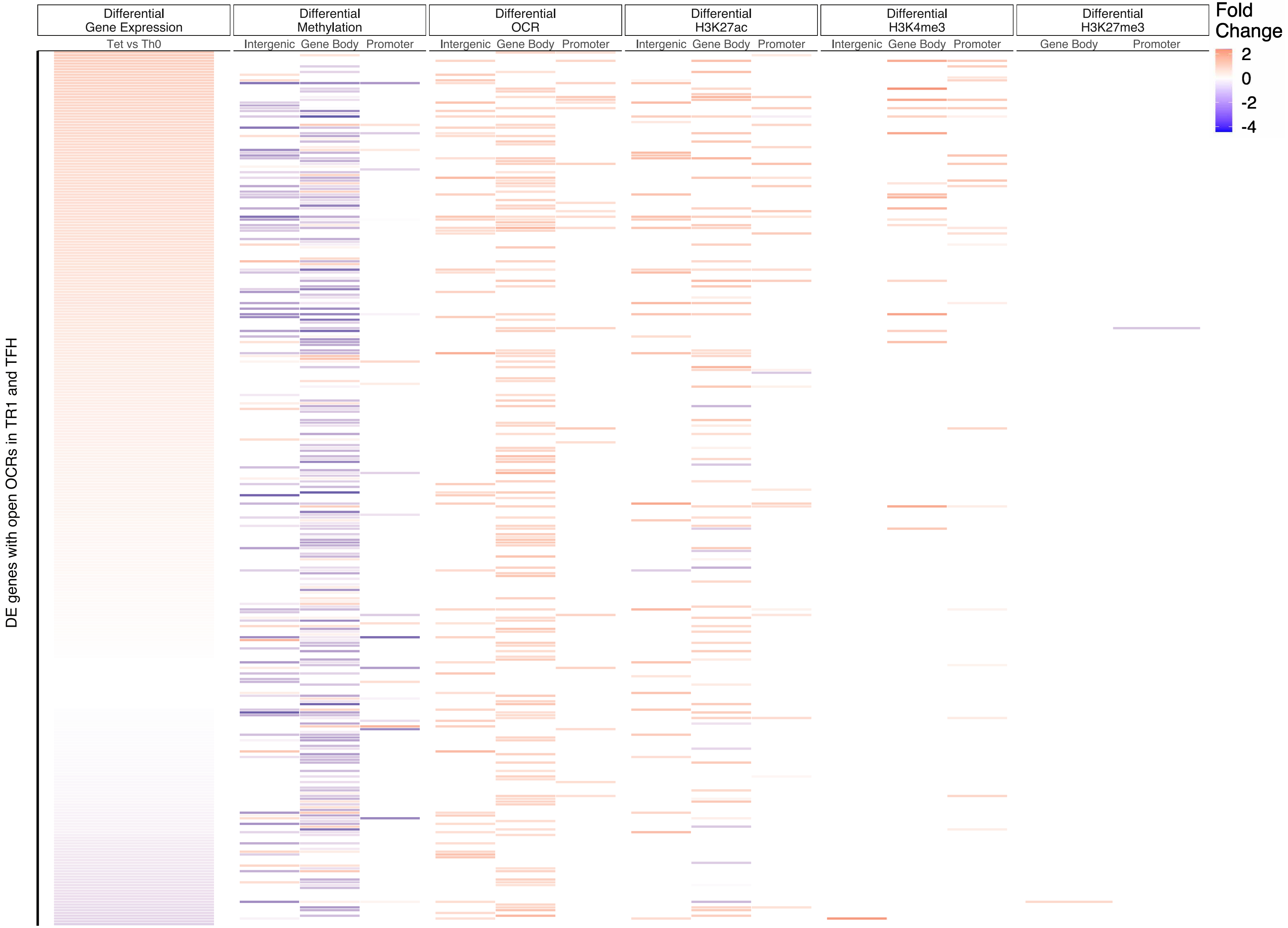
Differential Gene Expression
Tet vs Th0
Differential Methylation
Differential OCR
Differential H3K27ac
Differential H3K4me3
Differential H3K27me3
Intergenic
Gene Body
Promoter
Fold Change
2
0
-2
-4
DE genes with open OCRs in TR1 and TFH

C

Suppl. Fig. 2

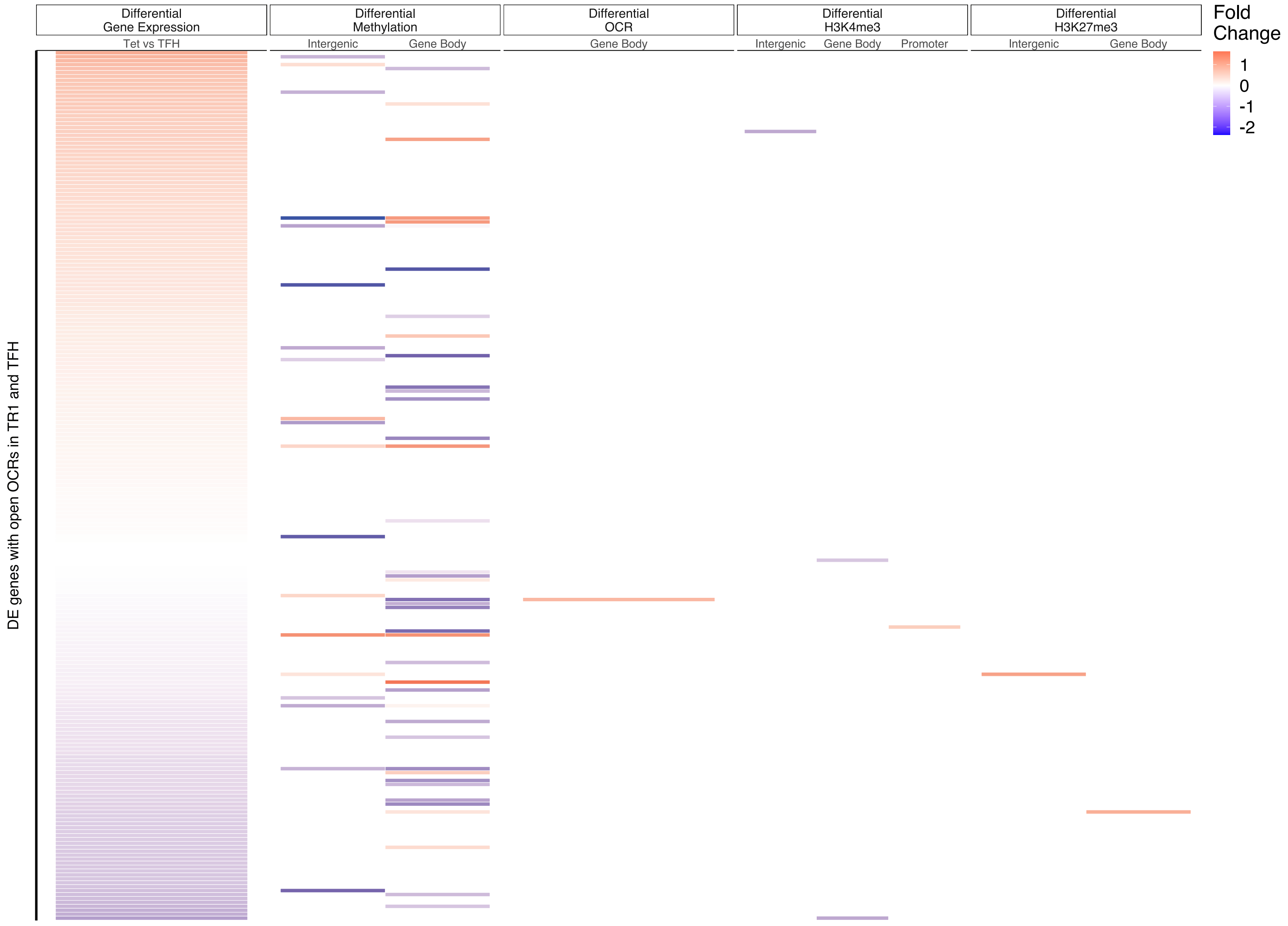

Differential Gene Expression
Differential Methylation
Differential OCR
Differential H3K4me3
Differential H3K27me3
Tet vs TFH
Intergenic
Gene Body
Gene Body
Intergenic
Gene Body
Promoter
Intergenic
Gene Body
DE genes with open OCRs in TR1 and TFH
Fold Change
1
0
-1
-2

Suppl. Fig. 2

D

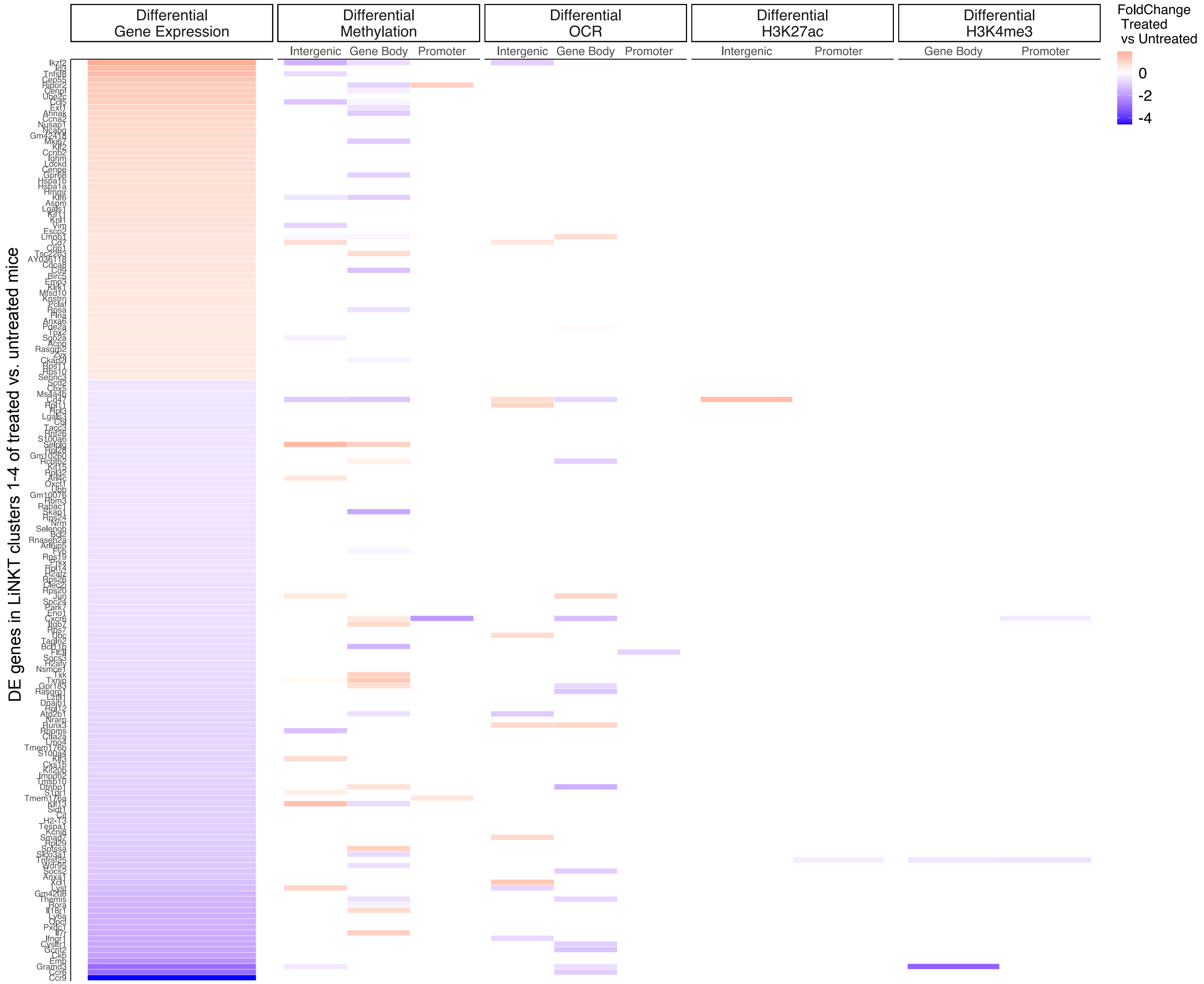
Differential Gene Expression
Differential Methylation
Intergenic
Gene Body
Promoter
Differential OCR
Intergenic
Gene Body
Promoter
Differential H3K27ac
Intergenic
Promoter
Differential H3K4me3
Gene Body
Promoter
FoldChange Treated vs Untreated
0
-2
-4
DE genes in LiNKT clusters 1-4 of treated vs. untreated mice

Supplementary Fig. 2. **Distribution of epigenetic modifications underpinning the re-programming of LiNKT cells in response to αGalCer/CD1d-NPs as compared to the Th0-TFH and Th0-TR1-like cell differentiation pathways.** Figure provides heatmaps displaying the specific types of epigenetic modifications associated with differential gene expression among the various T cell subsets studied herein.
